# Gene regulation and speciation in a migratory divide between songbirds

**DOI:** 10.1038/s41467-023-44352-2

**Published:** 2024-01-02

**Authors:** Matthew I. M. Louder, Hannah Justen, Abigail A. Kimmitt, Koedi S. Lawley, Leslie M. Turner, J. David Dickman, Kira E. Delmore

**Affiliations:** 1 Biology Department, Texas A&M University, College Station, TX USA; 2 Department of Veterinary Integrative Biosciences, School of Veterinary Medicine and Biomedical Sciences, Texas A&M University, College Station, TX USA; 3 Milner Centre for Evolution, Department of Biology & Biochemistry, University of Bath, Bath, UK; 4 Department of Neuroscience, Baylor College of Medicine, Houston, TX USA

**Keywords:** Evolutionary genetics, Behavioural genetics, Animal migration

## Abstract

Behavioral variation abounds in nature. This variation is important for adaptation and speciation, but its molecular basis remains elusive. Here, we use a hybrid zone between two subspecies of songbirds that differ in migration – an ecologically important and taxonomically widespread behavior---to gain insight into this topic. We measure gene expression in five brain regions. Differential expression between migratory states was dominated by circadian genes in all brain regions. The remaining patterns were largely brain-region specific. For example, expression differences between the subspecies that interact with migratory state likely help maintain reproductive isolation in this system and were documented in only three brain regions. Contrary to existing work on regulatory mechanisms underlying species-specific traits, two lines of evidence suggest that trans- (vs. cis) regulatory changes underlie these patterns – no evidence for allele-specific expression in hybrids and minimal associations between genomic differentiation and expression differences. Additional work with hybrids shows expression levels were often distinct (transgressive) from parental forms. Behavioral contrasts and functional enrichment analyses allowed us to connect these patterns to mitonuclear incompatibilities and compensatory responses to stress that could exacerbate selection on hybrids and contribute to speciation.

## Abstract (web summary)

Little is known about the genetic basis of many natural behaviours and how they contribute to speciation. Here the authors address this by identifying genes linked to migration of a songbird, investigating how these gene are regulated, and connecting them to potential barriers between species.

## Introduction

Considerable variation in behavioral traits exists in nature and is important for both adaptation and speciation. There is a strong genetic basis to many of these traits but major gaps in our understanding of behavioral genetics exist. For example, much of our knowledge comes from invertebrates and laboratory animals^1–3^. This focus arises in part from difficulties associated with quantifying behavioral traits in natural populations of vertebrates. The development and adoption of molecular tools has also been slower in these populations^4–6^ and ultimately means that our knowledge of behavioral genetics is confined to a small set of organisms and traits.

Both protein coding changes and alterations in gene regulation likely contribute to behavioral variation. Alterations in gene regulation could derive from cis- or trans-regulatory (i.e., proximate or distant) changes. This is a second gap in our understanding of behavioral genetics; only a handful of studies have attempted to distinguish between the roles of cis- versus trans-regulatory changes in generating behavioral variation. The studies that have been conducted often lacked context, using blood, whole brains and/or animals that were not engaging in the behavior of interest^7–10^. Regulatory divergence important for behavior is likely limited to specific brain regions and constitutive differences in expression may not relate directly to these behaviors^11,12^. Evolutionary constraints on cis- vs. trans-regulation likely differ (e.g., cis-regulatory changes may have fewer pleiotropic effects^13,14^) and thus, knowledge of their contribution to behavioral variation is important for understanding how behaviors evolve.

Behavioral genetics is relevant for our understanding of speciation. For example, behavioral traits could serve as both pre- and post-zygotic barriers to gene flow^15,16^. Most work focuses on their role as pre-zygotic isolating barriers, but they could also serve as extrinsic postzygotic isolating barriers, with hybrids exhibiting intermediate behaviors that fall outside parental niches^17,18^. We know very little about the molecular basis of extrinsic postzygotic isolating barriers^19^. There is also evidence for transgressive gene expression in hybrids (i.e., over or under-expression compared to parental forms). Transgressive expression is often assumed to derive from Dobzhansky-Muller incompatibilities, with recombination in hybrids uncoupling regulatory elements that co-evolved in parental forms^20,21^. These patterns could also be cellular responses to other forms of selection against hybrids but this connection is rarely examined^22^.

**Fig. 1:**
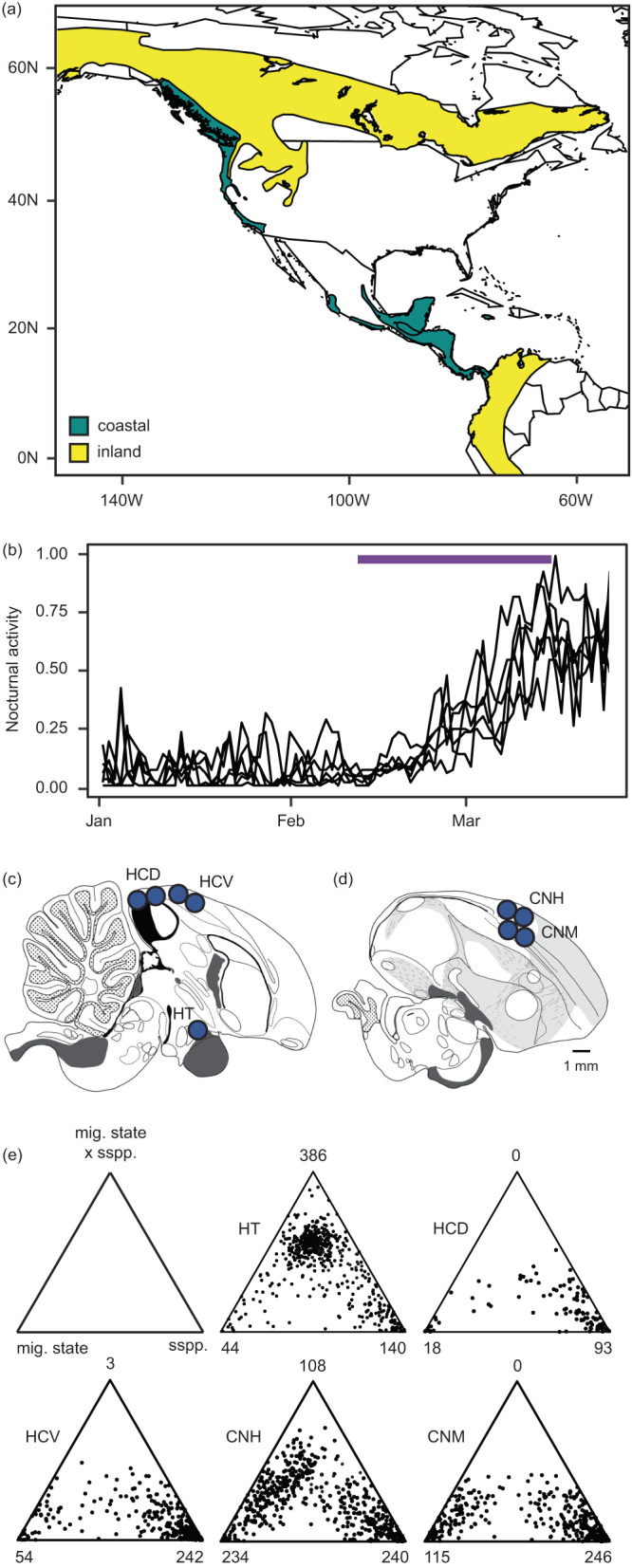
Study details. **a** Map showing ranges of coastal (teal) and inland (yellow) Swainson’s thrushes and origin of birds used in present study (star, hybrid population in Pemberton, British Columbia, Canada). **b** Nocturnal activity documented for birds sampled on spring migration. The period over which photoperiod and temperature were changed to simulate migratory behavior shown in purple. Birds were sampled during the winter (late Jan, non-migratory season) and spring migration (late Mar) seasons. **c**, **d** Histology of songbird brain, showing sagittal sections through the midline and 2 mm from the midline, respectively. Example brain punches used for sampling shown in blue (HT hypothalamus, HCD hippocampus dorsal, HCV hippocampus ventral, CNH Cluster N hyperpallium, CNM Cluster N mesopallium). **e** Ternary plots showing genes differentially expressed in at least one of three comparisons – between migratory states (environment), subspecies (genotype) and/or the interaction between these two levels (genotype x environment, GxE). Numbers of genes differentially expressed in each category shown at triangle edges, with first triangle indicating categories. Source data for panels b and e are provided as a Source Data file. Distribution map obtained from the BirdLife International and Handbook of the Birds of the World^106^ (http://datazone.birdlife.org/species/requestdis). Images of brains obtained from the Zebra Finch Atlas (http://www.zebrafinchatlas.org/gene_display/histological-atlas).

We used the Swainson’s thrush (*Catharus ustulatus*) to begin filling gaps in our knowledge of behavioral and speciation genetics. The Swainson’s thrush is a migratory songbird with two subspecies (coastal and inland). These subspecies form a narrow hybrid zone in western North America and take different routes on migration (Fig. 1a^23^). Direct tracking data showed that hybrids take intermediate routes on migration and ecological modelling showed that these routes are ecologically inferior to those of parental forms, suggesting differences in migration act as extrinsic postzygotic isolating barriers between thrushes^24,25^. The Swainson’s thrush has already been the focus of considerable genomic work, including a genome-wide association study identifying genomic regions underlying migratory orientation^26^ and genome scans identifying genomic regions under selection^27^. We will complement this genomic work with analyses of gene expression here.

Most songbird species migrate at night. If held under conditions that mimic seasonal changes they experience in nature, they will continue to transition between non-migratory and migratory states while in captivity; they become nocturnal and exhibit restless, directed behavior during the migratory period^28^. We capitalized on this behavioral assay and variation in the migratory behavior of thrushes here, comparing gene expression across migratory states, subspecies, and at the intersection between these levels (i.e., genes with subspecies x migratory state effects on expression, GxE genes). These GxE genes are of particular interest as they may capture variation in gene expression that is not only important for the transition between migratory states but also helps maintain subspecific differences between thrushes. We also integrated hybrids into our analyses, using allele-specific expression to distinguish between the effects of cis- and trans-regulatory changes on differential expression and examine patterns of transgressive expression in hybrids. We included a comparison across migratory states in analyses of transgressive expression. Migration is energetically costly^29,30^ and hybrid fitness can be both environmentally dependent and exacerbated by stress^31–34^. Accordingly, we expected to find greater transgressive expression in the migratory (vs. non-migratory) state.

In this work we identify genes differentially expressed between migratory states, subspecies and at the interaction between these levels. We use hybrids to show differential expression in the former genes is regulatory by changes in transregulatory regions and identify genes that are misexpressed in hybrids and could contribute to ecological speciation. There is considerable interest in the genetic basis of seasonal migration^35–37^. Transcriptomic work has been conducted but mostly focused on the transition between migratory states and signals from a small number of tissues (e.g., blood^38^ or whole brain^39,40^). We expanded this work in several ways, including subspecies, hybrids and multiple brain regions in our analyses. The inclusion of subspecies allowed us to examine the molecular basis of not only the transition between migratory states but also subspecies-level differences in this behavior. The integration of hybrids allowed us to distinguish between the effects of cis- and trans-regulatory changes using analyses of allele-specific expression. Hybrids also allowed us to examine patterns of transgressive expression, connecting gene expression to speciation.

## Results

Our study focused on juvenile male thrushes from three populations – parental coastal and inland populations adjacent to the hybrid zone (Vancouver and Kamloops, British Columbia, respectively; *n* = 9 birds/populations) and one hybrid population (Pemberton, British Columbia; *n* = 18 birds; Fig. 1a; Supplementary Data 1). Birds were captured at the end of the breeding season and brought into captivity where they were maintained in individual cages and under conditions (photoperiods and temperatures) that mimicked their natural environment. We used motion sensors to document the transition between migratory states (Fig. 1b; Ramirez et al. 2022) and sampled birds during the non-migratory (winter) and migratory (spring) states. We constructed RNAseq libraries for five brain regions: the hypothalamus, two regions of the hippocampus (dorsal and ventral) and two regions of Cluster N (in the hyperpallium and mesopallium; Fig. 1c, d). These brain regions are likely important for migration; the hypothalamus helps generate daily rhythms^41^, the hippocampus processes spatial information^42^ and Cluster N is a visual area of the brain that is activated during migration and is thought to processes geomagnetic information^43,44^.

### Differential expression between migratory states

**Fig. 2:**
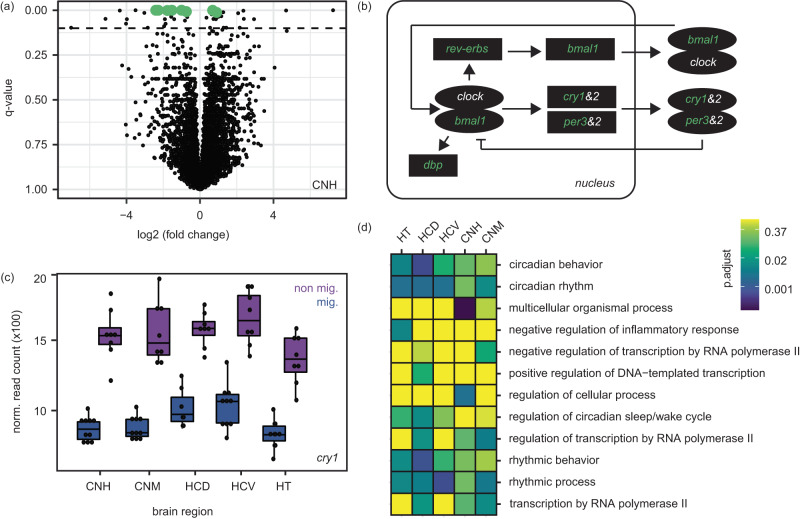
Results from comparison between migratory states. **a** Volcano plot showing differential expression between the migratory states, using results from the hyperpallium of Cluster N as an example. Dotted line shows q-value above which differential expression is considered significant. Green points identify genes differentially expressed in all brain regions, six of which are important components of the circadian clock. **b** Schematic of the molecular factors that regulate circadian patterns in birds (following Cassone 2014). Genes differentially expressed in all brain regions are shown in green. Positive elements CLOCK and BMAL1 enter the nucleus and activate expression of genes whose promoters contain an E-Box. Among these are the negative elements *period 2&3* and *cryptochromes 1&2*, *Rev-Erbs(α and β)* and *Dbp*. *Rev-Erbs* form a secondary loop regulating *Bmal1* transcription. The *pers* and *crys* are translated and reenter the nucleus to interfere with CLOCK/BMAL1 activation. **c** Example expression levels for one of the circadian genes that is differentially expressed across migratory states in all five brain regions (*n* = 18 birds; 8 non migratory and 10 migratory state). Boxplots show minimum, maximim, median, first and third quartiles. **d** Results from GO analyses including all categories significantly enriched in at least one brain region. FDR adjusted *p*-values from a cumulative hypergeometric test run in go:profiler are shown. HT hypothalamus, HCD hippocampus dorsal, HCV hippocampus ventral, CNH Cluster N hyperpallium, CNM Cluster N mesopallium. Source data for panels a and c are provided as a Source Data file.

We began our analysis with parental forms, identifying genes that were differentially expressed between the two migratory states. Depending on the brain region, between 18 and 234 genes were differentially expressed between these states (0.11–1.43% of genes; Fig. 1e; Supplementary Data 2). Eight genes were differentially expressed in all brain regions (*bmal1, cry1, dbp, dedd2, hsf2, rev-erbα, rev-erbβ*, and *per3*). Six of these genes have known associations with the circadian clock (Fig. 2a–c^45^). Gene ontologies related to the circadian clock and biological rhythms were also enriched in genes differentially expressed between migratory states in all brain regions (Fig. 2d).

We used a meta-analysis to test for overlap between the genes we identified in this study and previous work comparing gene expression across migratory states in birds. We limited our analysis to other unbiased, transcriptome-wide studies and ultimately identified seven studies and 4153 genes. 339 of these genes were associated with migration in two or more studies (Supplementary Data 3); only six overlapped with the genes we identified (Supplementary Data 2). This is not more genes than would be expected by chance. For example, the hyperpallium of Cluster N had four genes that overlapped with the list generated by our metanalysis. This was not different from the 4.85 genes predicted to overlap by chance alone (234/16,334 genes were differentially expressed between the seasons in this brain region and we had 339 genes [or “draws”] in our list of candidates).

### Differential expression between migratory states and subspecies

**Fig. 3:**
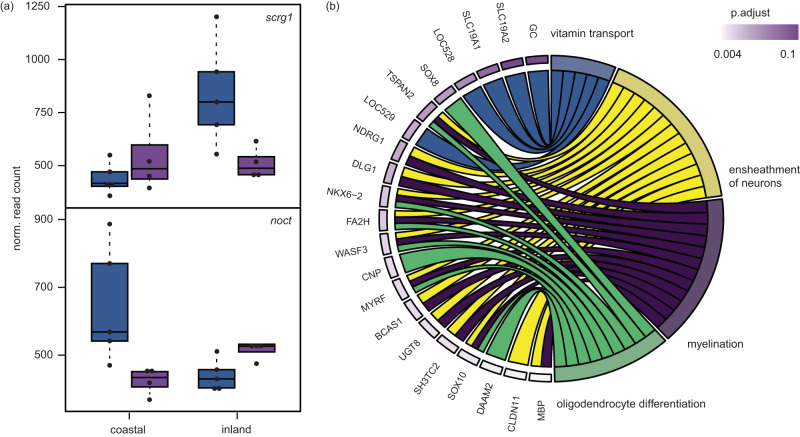
Results from the comparison between seasons and subspecies. **a** Example expression levels from two genes showing GxE patterns of differential expression in the hypothalamus (n = 18 birds; 4 coastal non-migratory, 4 inland non-migratory, 5 coastal migratory, 5 inland migratory; boxplots show minimum, maximum, median, first and third quartiles). **b** Results from GO analyses using GxE genes in the hypothalamus. Genes and GO categories they are enriched for are shown. Genes are ordered by their *p*-values from differential expression analyses. FDR-adjusted *p*-values from a cumulative hypergeometric test run in go:profiler are shown. The figure is limited to GOs from Biological Processes; remaining ontologies enriched in these and other brain regions can be found in Supplementary Data 5. Source data are provided as a Source Data file.

Depending on the brain region, between 93 and 246 genes were differentially expressed between the subspecies (0.56–1.47% of genes; Fig. 1e). These genes are constitutively differentially expressed between the subspecies. We are more interested in genes with subspecies x migratory state (GxE) effects on gene expression as they likely underlie differences in migration (or carry-over effects from the non-migratory state) that help maintain subspecies differences between thrushes. Only three brain regions included genes with GxE effects on expression: the hypothalamus (*n* = 386 [2.3% of genes]), the Cluster N region of the hyperpallium (*n* = 108 [0.6% of genes]), and a very small number in the ventral portion of the hippocampus (*n* = 3 [0.02% of genes]; Figs. 1e; 3a; Supplementary Data 4).

Several gene ontologies were enriched in GxE genes from the hypothalamus and the hyperpallium of Cluster N. For example, ontologies falling under the parent term “nervous system development” were enriched in the hypothalamus and included “ensheathment of neurons”, “myelination” and “oligodendrocyte differentiation”. Ontologies important for energy production and transport were also enriched in both brain regions and included ‘signaling receptor activity’, ‘monoatomic ion transport’, ‘vitamin transport’, and ‘proton motive force-driven ATP synthesis’) (Supplementary Data 5).

We found limited overlap between previous genomic work with Swainson’s thrushes and the GxE genes identified here. Recall, Delmore et al.^26^ mapped migratory orientation in this species and Delmore et al.^27^ identified genomic regions under selection. None of the GxE genes we identified here overlapped with previous mapping results and only 18/386 and 3/108 GxE genes from the hypothalamus and Cluster N, respectively, overlapped with the selection scan (Supplementary Data 4). Twelve and three genes (in the hypothalamus and Cluster N, respectively) were expected to overlap between our dataset and the selection scan by chance (e.g., 386/16221 genes are GxE in the hypothalamus and 517 genes showed signatures of selection in Delmore^27^).

### Regulatory mechanisms underlying differential expression

The expression differences we documented between subspecies (with or without an interaction with migratory state) could derive from changes in cis- or trans-regulation. We used two approaches to distinguish between these forms of regulation, starting with hybrids and allele-specific expression (ASE).

Cis-regulatory sequences are proximate to genes and thus, are expected to drive differences in the abundance of the two possible alleles in hybrids. This is not the case for trans-regulatory factors as they are distant from genes and should impact the expression of both alleles in hybrids^20^. We limited our ASE analyses to genes where at least three hybrid individuals were heterozygous for subspecies-specific alleles and then calculated the log2 fold changes between coastal and inland alleles at each gene. Very few genes showed significant evidence of ASE. As we are more interested in understanding the broader evolutionary mechanisms than identifying specific ASE candidate genes, we compared log2 fold changes between alleles in hybrids and differential expression between subspecies. Correlations across hybrid allele-specific expression and expression in parental forms are expected if divergent cis-regulatory regions control differences in gene expression^10,20^.

**Fig. 4:**
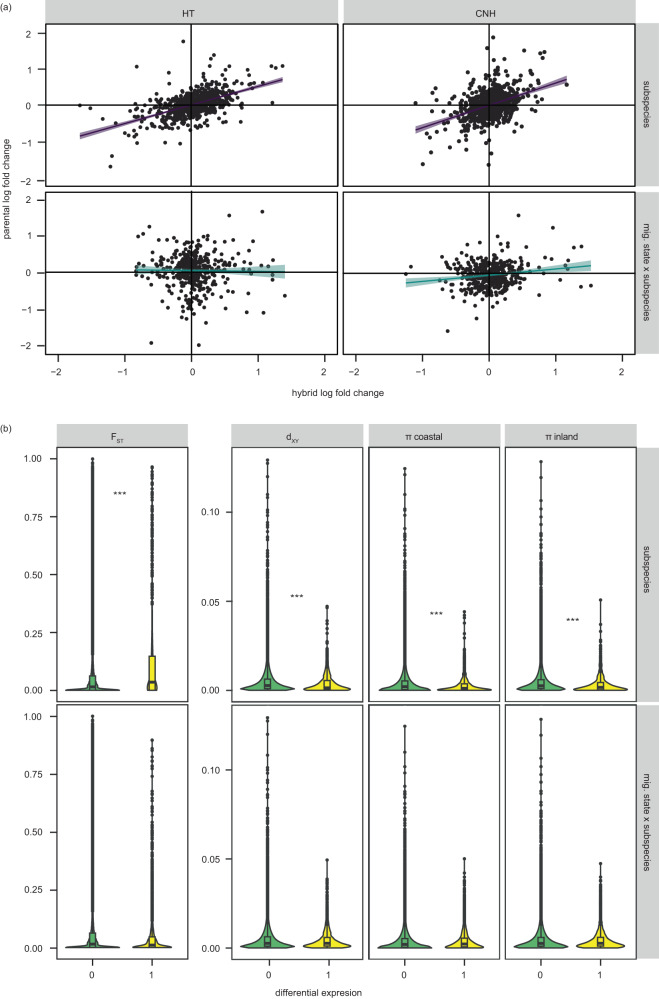
Regulatory mechanisms underlying differential expression. **a** Results from analyses of allele-specific expression. Relative expression (log2 fold changes) between coastal and inland alleles in hybrids is compared to relative expression between parental forms in top panels. The contrast between migratory states is added to the bottom panels, comparing fold changes between migratory states in hybrids to fold changes between migratory states in parental forms (i.e., GxE expression). Results from the hypothalamus (left panels, HT) and hyperpallium of Cluster N (right panels, CNH) are shown. Prediction line and 95% confidence band from linear models are shown. **b** Relationship between genomic variation estimated in CNEs and introns and gene expression (between subspecies and with or without an interaction with migratory state), including estimates of F_ST_ and d_XY_ between coastal and inland forms of Swainson’s thrushes and nucleotide diversity (π) within the subspecies. (0 = not differentially expressed; 1 = differentially expressed; *** *p*-value < 0.0001 [exact *p*-values can be found in Supplementary Data S6] derived from linear models run with genomic variation as the response variable, differential expression as the predictor variable and gene as a random effect; *n* = 13009 genes in top four panels and 14605 genes in bottom four panels). Boxplots show median, first and third quartiles. Source data for all panels are provided as a Source Data file.

We tested for ASE in two comparisons between hybrids and parental forms. First, we compared changes in expression (log2 fold changes) at subspecies-specific alleles in hybrids with log2 fold changes between parental forms (regardless of migratory state). We then compared log2 fold changes of subspecies-specific alleles between migratory states in hybrids $\left(\log 2\left(\frac{{coastal\_allele\_nonmig}}{{coastal\_allele\_mig}}/\frac{{inland\_allele\_nonmig}}{{inland\_allele\_mig}}\right)\right)$ against fold changes of parental forms between migratory states $\left(\log 2\left(\frac{{coastal\_nonmig}}{{coastal\_mig}}/\frac{{inland\_nonmig}}{{inland\_mig}}\right)\right)$ (i.e., GxE expression). We focused on the two brain regions with the strongest patterns of GxE expression, the hypothalamus and the hyperpallium of Cluster N. Differential expression between the subspecies regardless of migratory state appears to be controlled in large part by cis-regulatory changes. Specifically, log2 allelic fold changes between subspecies-specific alleles in hybrids were correlated with log2 fold changes between parental forms in both brain regions (hypothalamus: R^2^ = 0.17, *n* = 909, *p*-value < 0.0001; hyperpallium of Cluster N: R^2^ = 0.3, *n* = 908, *p*-value < 0.0001; Fig. 4a). This was not the case for tests of ASE between subspecies-specific alleles and migratory state (i.e., GxE expression). Log2 allelic fold changes were not correlated across hybrid and parental forms in the hypothalamus (R^2^ = −0.002, *n* = 415, *p*-value = 0.57) and they were only weakly correlated in the hyperpallium of Cluster N (R^2^ = 0.009, *n* = 428, *p*-value = 0.03; Fig. 4a), suggesting differential expression for GxE expression at these genes is predominantly due to changes in trans-regulatory mechanisms. Results from an alternate approach for estimating ASE can also be found in the Methods.

ASE analyses are traditionally applied to F1 hybrids, where individuals have precisely one haplotype from each parental species. We cannot be sure that is the case in our hybrids as they come from a natural hybrid zone and are not all F1s (Supplementary Fig. 1). Nevertheless, it is telling that we uncovered different patterns with and without migratory state (indicating we can uncover cis-regulatory changes with our dataset). In addition, we obtained similar results in a second analysis using estimates of genomic variation. We expand on this analysis below.

Expression QTLs (eQTLs) have been used to distinguish between cis- and trans-regulatory differences, with authors looking for associations between genetic variation and patterns of differential gene expression. Close (proximate) eQTL are considered cis and those further away (distal) are considered trans^46^. We did not have a large enough sample size to identify eQTL here, but we used a similar principle. Specifically, we obtained whole genome resequencing data for 15 individuals/subspecies and estimated four population genetic parameters at conserved non-coding elements (CNEs) proximate to each gene (+/−10,000 bp) and in introns that likely harbor cis-regulatory regions including promoters, enhancers and silencers): F_ST_ (relative differentiation) and d_XY_ (pairwise sequence divergence) between the subspecies and π (nucleotide diversity) within each subspecies. Previous work has found correlations between genomic variation and expression divergence^22,47,48^ but---following the principle described for eQTL---these correlations are only expected when differences in gene expression derive from cis-(proximate) regulatory changes. In support of this suggestion and results from our ASE analyses, F_ST_ was elevated at genes that were constitutively differentially expressed between the subspecies and the remaining three parameters (d_XY_ and π within coastal and inland populations) were reduced (Fig. 4b; Supplementary Data 6, all *p*-values < 0.000005). These patterns were consistent across regions and contrasted with results at GxE genes. Specifically, we did not find an association between any of our population genetic parameters and differential expression at GxE genes in the hyperpallium of Cluster N (F_ST_: estimate = −0.025, χ^2^ = 2.61, *p*-value = 0.11; d_XY_: estimate = −0.0042, χ^2^ = 0.72, *p* = 0.40; π coastal: estimate = −0.001, χ^2^ = 0.064, *p* = 0.80; π inland: estimate = −0.0031, χ^2^ = 0.29, *p* = 0.59) or the hypothalamus (Fig. 4b; F_ST_: estimate = −0.012, χ^2^ = 2.00, *p*-value = 0.16; d_XY_: estimate = 0.00060, χ^2^ = 0.050, *p* = 0.83; π coastal: estimate = 0.0017, χ^2^ = 0.22, *p* = 0.64; π inland: estimate = 0.0018, χ^2^ = 0.29, *p* = 0.59; Fig. 4b).

### Transgressive expression in hybrids

**Fig. 5:**
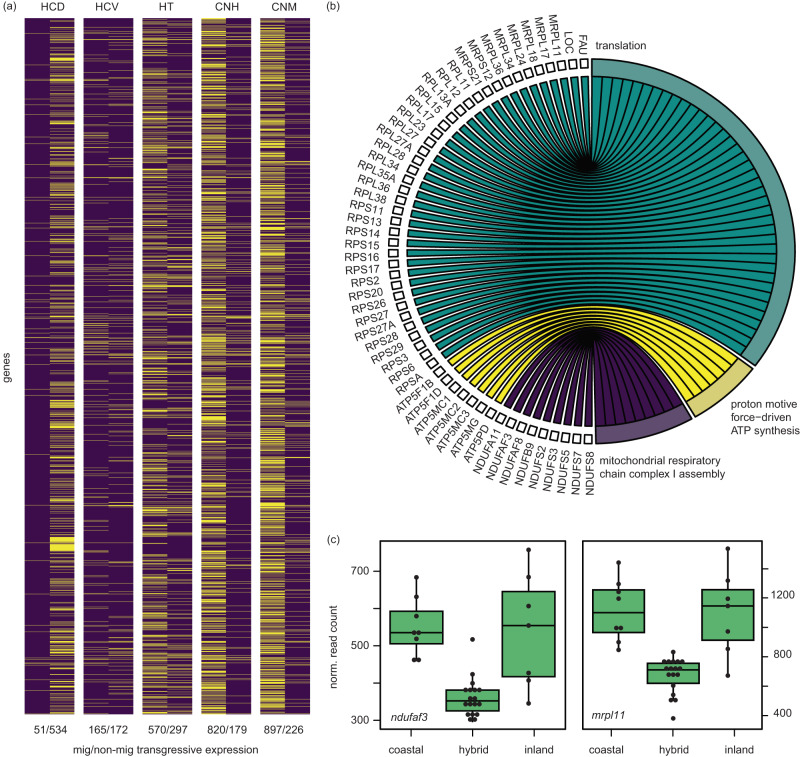
Results from analyses of transgressive expression in hybrids. **a** Heatmap limited to genes exhibiting transgressive expression in at least one brain region and migratory state. Genes that were transgressively expressed are shown in yellow. Numbers below the heatmap show how many genes were transgressively expressed in each brain region and condition (# transgressive during non-migratory state / # transgressive during migratory state). **b** Results from GO analyses using genes underexpressed in HCD during the non-migratory state. The top three GO categories from Biological Processes are shown; remaining ontologies enriched in these and other brain regions can be found in Supplementary Data 5. **c** Example patterns of transgressive gene expression at two genes in the HT, including one gene important for energy production in the mitochondria (NDUFA3) and one that is transcribed in the nucleus but forms part of the mitochondrial ribosome (MRPL11; *n* = 33 birds, 7 coastal, 8 inland and 18 hybrid). HT hypothalamus, HCD hippocampus dorsal, HCV hippocampus ventral, CNH Cluster N hyperpallium, CNM Cluster N mesopallium. Boxplots show minimum, maximim, median, first and third quartiles. Source data for panels **a** and **c** are provided as a Source Data file.

In our last set of analyses, we examined transgressive expression in hybrids (i.e., over or under-expression compared to parental forms). Transgressive expression was more common than differential expression (by migratory state and/or subspecies); depending on the brain region, between 331 and 951 genes exhibited transgressive patterns in hybrids (2.1–5.9% of genes; Fig. 5a). There was limited overlap between genes showing transgressive expression in hybrids and those differentially expressed between the subspecies (e.g., focusing on the hyperpallium of Cluster N, only 6/875 transgressive genes were also differentially expressed between the subspecies).

We predicted that transgressive expression would be more common in the migratory state because migration is energetically costly and hybrid fitness can be both environmentally dependent and exacerbated by stress. In support of this prediction, three out of five brain regions exhibited far more transgressive expression during the migratory state (the hypothalamus and both regions of Cluster N; Fig. 5a). Surprisingly, transgressive expression in one brain region (the dorsal portion of the hippocampus) was more common during the non-migratory period. We expand on these two patterns below.

Focusing on increased transgressive expression in the migratory states, we divided genes into those that were over vs. underexpressed and tested for functional enrichment in each brain region. Several ontologies were enriched in these gene sets, including ‘ubiquitin-dependent protein catabolic process’ in genes overexpressed in the mesopallium of Cluster N and ‘Rab protein signal transduction’ in genes overexpressed in the hypothalamus (Supplementary Data 5). Ubiquitin and Rab proteins are important for cellular stress responses. Ubiquitin tags damaged and misfolded proteins for degradation following a number of stimuli and stress conditions^49–51^. Rab proteins regulate intracellular membrane trafficking, facilitating processes like autophagy and responding to immune system modulators^52–54^.

Moving on to the observation of greater transgressive expression in the non-migratory state and the dorsal portion of the hippocampus, ontologies related to the mitochondrial respiratory chain (e.g., ‘mitochondrial respiratory chain complex’, ‘NADH dehydrogenase activity’) and nuclear-encoded ribosomal proteins that are imported into the mitochondria (*mrp*s) were enriched in genes that were underexpressed in this brain region (Fig. 5b, c; Supplementary Data 5). Ontologies related to translation and the control of gene expression (e.g., ‘post-translational control of aberrant gene expression’) were enriched in overexpressed genes of this brain region.

## Discussion

Seasonal migration is a complex, and ecologically important behavior that contributes to speciation. We leveraged variation in the migratory behavior of Swainson’s thrushes to examine patterns of gene expression linked to migration and subspecies-specific differences in this trait. Our study design (e.g., use of multiple brain regions, behavioral and taxonomic contrasts) allowed us to gain unique inference in the genetic basis of both behavioral traits and speciation, including the mechanistic role that gene expression plays in speciation.

The transition between migratory states and subspecies-specific differences in this behavioral trait are clearly governed by multiple genes. Circadian genes and related ontologies dominated the transition between migratory states, supporting previous work on the genetics of migration^35,37^. Circadian rhythms in peripheral tissues are thought to be controlled by master regulators in the hypothalamic suprachiasmatic nuclei, pineal gland, and retina^45,55^. Our observation that all brain regions showed common patterns for circadian genes falls in line with the idea. Beyond circadian genes, we documented limited overlap with genes differentially expressed between migratory states in other studies. Migration has long been predicted to have a common genetic basis across birds^35,36^. Differences in study design (e.g., age, sex and timing of sampling) likely affected our ability to find overlap but for now, our results suggest that beyond circadian genes, the propensity to migrate is not controlled by the same genes. This does not preclude the possibility that the same pathways control this behavior^56^. Future work using consistent study design (including bioinformatic analyses [e.g., careful consideration is needed when conducting functional analyses^57^]) will continue to inform this question, testing if the same pathways control the transition between migratory states or if this transition is facilitated by different molecular mechanisms. Within thrushes, it will be valuable to conduct the same experiment focusing on fall (vs. spring) migration, to test if the same genes are involved in the transition between states and if any differences exist between these two legs of migration. Inland birds take different routes on fall and spring migration suggesting gene expression may differ between legs in this subspecies (Delmore et al.^23^). Johnston et al.^58^ assayed gene expression during fall migration in thrushes and noted the importance of circadian genes in the transition between states but differences in study design (e.g., the timing of sampling and tissues assayed) preclude a formal comparison.

Genes with subspecies x migratory state effects on expression (GxE genes) were limited to just three of the five brain regions examined and were enriched for ontologies related to nervous system development. There is already evidence that nervous system development is important for migration. Brain size and neuron numbers are larger in migrants and authors have suggested that birds prepare for migration by building new neurons and connections that will facilitate processes like memory acquisition and spatial orientation^59,60^. Our results suggest that neural development could also be important for generating subspecies-specific differences in the migratory behavior of thrushes, driving evolutionary divergence in behavior and facilitating speciation. Several additional ontologies and the genes within them deserve additional attention in the future as well. For example, ‘signaling receptor activity’ was enriched in the hypothalamus. Genes with this ontology could be important for the night activation of Cluster N and subspecies specific-differences in orientation. Cluster N is thought to processes geomagnetic information in songbirds^43,44^. It is connected to the retina through the thalamofugal pathway and relies on low levels of light at dusk to function^43,61^. The genes we identified here may help relay information between the retina and Cluster N and allow thrushes to both perceive the magnetic field and make different decisions about where to migrate based on this information.

We documented limited overlap between GxE genes and previous genomic work in the Swainson’s thrush. This was the first indication that differential expression at GxE genes may be regulated by changes in trans-regulation and was supported by both allele-specific expression analyses in hybrids and new genomic data generated for the present study. The general sentiment in the literature is that variation in morphological traits is controlled by cis-regulatory changes^4,20^ and there is growing evidence for the same pattern in behavioral traits^7–10^. In the case of behavioral traits at least, this pattern may reflect limitations in study design as most behavioral work on this topic used whole brain tissue and/or failed to include a behavioral contrast. We would not have identified trans-regulatory changes as major contributors to behavioral differences between the subspecies in our analysis without those additional layers (e.g., if we had just focused on genes differentially expressed between the subspecies regardless of season or if we had not included the hypothalamus and hyperpallium of Cluster N in our analyses). Trans-regulatory changes may be more important for behavioral traits because these traits likely rely on external cues for expression and trans-acting regulatory differences can respond more readily to these cues than cis-regulatory differences^62^. Migratory behavior is highly labile and can respond rapidly to changes in selection^63,64^. It has been suggested that genetic correlations across traits help facilitate this flexibility^63^. Trans-regulatory divergence may underlie these correlations, with a subset of genes serving as master regulators of multiple migratory traits. It will be of great interest to continue probing this pattern of trans-regulatory divergence in the future (e.g., using eQTL analyses to identify master regulators^65,66^ and additional molecular tools to narrow down the potential list of candidate genes^6^).

Note, we used our analyses of genomic variation to help distinguish between the effects of cis- and trans-regulatory changes but findings from these analyses also speak to the evolutionary processes that affect gene expression in Swainson’s thrushes. Recall, F_ST_ was elevated at genes differentially expressed between the subspecies regardless of migratory state; d_XY_ and π were reduced. At first glance, it may seem counterintuitive that F_ST_ and d_XY_ (two estimates of differentiation) exhibit different patterns at these genes. Nevertheless, it is important to keep in mind that F_ST_ is a relative measure of differentiation that includes a term for within population variation. d_XY_ does not include this term and, as a result, it often exhibits different patterns than F_ST_. In the case of thrushes and other taxa that have undergone periods of allopatry, background selection and recurrent selective sweeps in ancestral populations are thought to keep d_XY_ low, continually removing genetic variation from these regions^27,67–69^. Accordingly, the patterns we documented here suggest that genes differentially expressed between the subspecies regardless of migratory state have been experiencing strong selection (background and/or selective sweeps) throughout the geographic history of thrushes.

We end with a discussion of results from our analyses of transgressive expression. We documented considerable transgressive expression in hybrids. These patterns were strongly context-dependent. For example, as we predicted, transgressive expression was more common in the migratory state for three brain regions. Transgressive expression is often assumed to derive from negative epistatic interactions between regulatory elements in hybrids^70,71^ but these patterns may also reflect cellular attempts to compensate for other forms of selection in hybrids^22^. Functional analyses support the latter idea in thrushes, with ontologies related to cellular stress response being enriched in genes that were overexpressed in the migratory state. Future work controlling for cell types and connecting these patterns to hybrid fitness^72^ are needed but these findings could have interesting implications for speciation. For example, these compensatory mechanisms may fail to restore hybrid fitness and/or exacerbate problems in hybrids^22^. Differences in migration are considered extrinsic, postzygotic barriers to gene flow. Our results could take this form of reproductive isolation further, with conditions birds experience during migration uncovering and/or exacerbating fitness costs hybrids are already experiencing. Similar environment- or stress-related reductions in fitness have been documented in other systems^31–34^.

Contrary to our predictions, we documented increased transgressive expression during the non-migratory state in the hippocampus. Functional analyses suggested that these patterns derive from incompatibilities between mitochondrial and nuclear genomes in hybrids. Similar results have been reported in other studies^22,73–76^ and these findings could open an exciting new area of research in thrushes. Specifically, there is considerable interest in the role mitonuclear incompatibilities play in speciation^77,78^. These incompatibilities have not been examined directly in Swainson’s thrushes, but a steep geographic cline exists in the mitochondrial genome and is displaced from the nuclear genome^79^. In addition, reductions in the cognitive abilities of hybrid thrushes have been documented and are limited to the non-migratory state^80^. Transgressive expression could help explain this pattern; the hippocampus is important for learning and memory^81^. Reductions in the cognitive abilities of hybrids could represent the downstream effects of transgressive expression (and mitonuclear incompatibilities) in this brain region^82^. Studies of transgressive expression rarely include behavioral contrasts; most work is limited to constitutive patterns^70,76,83^ or comparisons between sterile and fertile hybrids^73,84^. Our study highlights the role context-dependent transgressive expression could play in speciation and we look forward to future work on this connection in the Swainson’s thrush and other systems.

## Methods

### Bird capture and housing

All experiments were performed in accordance with relevant guidelines and regulations. Protocols were approved by the Institutional Animal Care and Use Committee at Texas A&M (IACUC 2019-0066) and permits were obtained from Environment and Climate Change Canada (10921; SC-BC-2019-0016; SC-BC-2020-0016), the U.S. Fish and Wildlife Service (MB49986D-0; LE51239D-0), Texas Parks and Wildlife Commission (SPR-0419-067) and the United States Department of Agriculture (RIV19148289; RIV20152472).

Male juvenile thrushes were caught with mist nets and song playback in the coastal (Vancouver, BC, −123.2115, 49.2480), hybrid (Pemberton, BC, 50.26474,−122.867) and inland (Kamloops, BC, 50.9039, −120.3131) range. Males from parental ranges were captured in Aug 2020 (*n* = 9/subspecies) and hybrids in Aug 2020 and 2021 (*n* = 9 in 2020 and 9 in 2021; Supplementary Data 1). The hybrid population comprises ~40% hybrids^79^. We genotyped hybrids in the field using three RFLPs diagnostic of inland and coastal subspecies^24^ and kept birds with the highest degree of admixture. Ancestry could range from 0 (coastal, all three RFLPs are homozygous for coastal alleles) to 1 (inland, all three RFLPs are homozygous for inland alleles). Average ancestry of birds included in the experiment was 0.50 (range 0.17–0.83). Males were subsequently sequenced with a whole genome resequencing approach and ancestry as described below in “Genomic composition of hybrids”.

All birds were relocated to an animal facility at Texas A&M University where they were housed in individual cages and fed a diet consisting of berries, mealworms, egg, crackers, cottage cheese and red meat ad libitum^58^. We used previous data collected from free-flying hybrids fitted with geolocators to adjust the photoperiod experienced by captive birds to mimic natural conditions^23,24^. Specifically, we gradually decreased the photoperiod from the migratory state of 25 degrees Celsius and 14 h of light (L):10 h of dark (D) to the non-migratory state of 22 degrees Celsius and 11 L:13D in September– October. Birds were held in this non-migratory state until February-March when the photoperiod was gradually increased to 16 L:8D, mimicking spring migration and eventual arrival on the breeding grounds.

### Migratory behavior

We monitored migratory restlessness (*zugunruhe*) of birds using motion sensors designed at Texas A&M University^85^. Each cage was equipped with a AM312 Passive Infrared (PIR) sensor (HiLetGo, Guangdong, China) that detected any movement of the individual. All PIR sensors were powered by a protoboard and wired to a central unit using a Data Acquisition Card (DAQ). The DAQ interfaced the motion detections to the LabVIEW program on a central Windows Operating System computer. Data was output into a CSV file in 10-min increments. We validated the reliability of motion sensors by collecting behavioral data from a subset of birds (*n* = 21) using infrared (IR) cameras (D-link, DCS-932L Day/Night Network Surveillance Camera). We found that data from PIR sensors and IR cameras were highly correlated^85^.

We classified birds as non-migratory and migratory based on similar criteria to Johnston^58^ and Owen and Moore^86^. Specifically, the PIR sensors have a three-second delay after the detection of motion, so we divided motion detection records by three to estimate a more accurate movement count. We defined time increments as active when a bird moved greater than 20 times per 10 min. The number of increments per day was adjusted according to the photoperiod. Birds were classified as non-migratory when they were active for less than 5% of nightly time increments and migratory when active for greater than 40% of nightly time increments. Example plots of nocturnal behavior can be found in Fig. 1b.

### Tissue collection

We euthanized four birds/parental subspecies (coastal, inland and hybrid) during the non-migratory season and five birds/parental subspecies during the spring migratory season by decapitation. Twice as many hybrids were euthanized in each season (8 during the non-migratory period and 10 during the non-migratory period). Tissue was collected at night, 1-h following the onset of darkness in the subject room and over no more than three hours (3−4 individuals processed/day). Non-migratory birds were euthanized between January 20 and February 2 and migratory birds between Mar 28 and April 15. All birds exhibited desired behaviors for at least ten days prior to and the night of the euthanasia. We performed dissections under red light to prevent potential effects of white light on gene expression associated with circadian rhythms. Brains were dissected immediately after decapitation and frozen in isopentane cooled on dry ice to prevent tissue fracturing that can occur from rapid freezing (time between decapitation and freezing ranged from 3.5–8.3 min). We stored brains in liquid nitrogen until they could be transferred to a −80 °C freezer for long-term storage.

We bisected brains and sectioned the left hemisphere into 100 μm-thick sections using a CM1850 cryostat (Leica Biosystems, Buffalo Grove, IL, USA). A 1-mm disposable biopsy punch (Integra, York, PA, USA) was used to extract tissue from five brain regions: the ventral hypothalamus (one punch/section for 4–6 slices/bird), dorsal and ventral regions of the hippocampus (two punches/region/section for 5–7 slices/bird), and two regions of Cluster N (in the hyper- and mesopallium, two punches/region/splice for 5–8 slices/bird; Fig. 1c, d). We used the Zebra Finch Atlas for reference (http://www.zebrafinchatlas.org/gene_display/histological-atlas) when taking punches. Starting with the hypothalamus, we took serial punches from the midline to where the tractus septomesencephalicus begins to bifurcate and the cerebellum is no longer present. Punches were restricted to the medial ventral portions of the anterior hypothalamus. These punches were taken caudal to the rostral border of the optic chiasm, just medial to the lateral portion of the supraoptic nucleus and rostral to the ventral supraoptic decussation. Sampled areas included regions of the suprachiasmatic nuclei and the dorsal supraoptic decussation. Continuing with the hippocampus, we took four serial punches beginning just rostral to the median junction between telencephalon and cerebellum^87^ extending approximately 2.5 mm rostral of this region, such that most of the hippocampus was sampled. Cluster N was isolated approximately 2 mm laterally from the midline and ~1.5 mm rostral to the most rostral portion of the lateral ventricle. Cluster N is a large region which extends ~1.5 mm dorsoventral, ~1.5 mm mediolateral, and ~1 mm rostrocaudal^43,44^. We collected punches from a more rostral portion of the hyperpallium and just caudal to the initial punch. We also sampled the mesopallial region of Cluster N ventrally to the punches placed in the hyperpallium. Tissue punches were transferred to a −80 °C freezer until RNA extraction.

### RNA extraction, library construction and sequencing

All tissue punches were homogenized with a Pellet pestel cordless motor (Sigma Aldrich, St. Louis, MO, USA) and total RNA was extracted with Qiagen’s RNeasy Lipid Tissue Mini Kit. We confirmed RNA quality for all samples using a Tape Station and High Sensitivity RNA ScreenTape (Agilent, Santa Clara, CA; all RIN scores > 8). RNAseq libraries were prepared by Texas A&M’s Institute for Genomic Sciences and Society using TruSeq’s Stranded mRNA library prep and sequenced on a NovaSeq 6000 (paired-end 150 bp reads). The number of reads generated and mapped to the reference can be found in Supplementary Data S1.

### Read mapping and differential expression

We used the reference genome for the inland Swainson’s thrush in all bioinformatic analyses involving RNAseq data^88^. This reference genome was annotated using NCBI’s Eukaryotic Genome Annotation Pipeline (https://www.ncbi.nlm.nih.gov/genome/annotation_euk/Catharus_ustulatus/100) and RNAseq data from the brain, testes and ovaries. The final annotation (GCF_009819885.1) includes 19, 270 genes. We first removed adapters from RNAseq reads with Trim Galore! v0.3.7 (https://github.com/FelixKrueger/TrimGalore). To minimize mapping bias between the two subspecies, we used whole genome resequencing data from parental populations to identify SNPs (nearly) fixed for alternate alleles in the two subspecies and masked these regions. F_ST_ was estimated using vcftools 0.1.16 and SNPs were considered nearly fixed if they had values > 0.90 (lower the threshold for masking SNPs [e.g., those with F_ST_ values > 0.1] did not affect the number of reads that mapped to our reference genome, *p*-value = 0.99 and we did not document any bias towards the reference genome [inland subspecies] in the number of subspecies-specific SNPs or expression). Details on whole genome resequencing data and analyses can be found under ‘Sequence divergence’ below. We aligned all RNAseq samples to the masked genome using STAR (version 2.7^89^), using 2-pass alignment mode. We quantified read counts using HTSeq-count 0.11.2^90^. After removing genes with low expression (<1 count per million in at least 4 subjects), we normalized for read-depth and tested for differential expression of genes with DESeq2^91^. Differential expression models included migratory state (non-migratory vs. migratory), population, and an interaction term. Furthermore, given that some hybrid samples were extracted and sequenced in two batches, we included “batch” as a covariate to all expression models. Genes with an FDR-adjusted *p*-value < 0.10 were considered differentially expressed. Genes were considered transgressive in hybrids (i.e., misexpressed) when significantly differentially expressed for both comparisons of hybrid vs coastal and hybrid vs inland and also if expression levels were outside the range of parental populations (i.e., underexpressed if expression is significantly lower than each parental form; overexpressed if significantly higher than each parental form).

### Allele-specific expression

We tested for biased gene expression of parental alleles in hybrids (i.e., allele-specific expression). First, we followed GATK 4.2.0.0 best practices for variant calling on RNAseq reads, which includes reformatting alignments that span introns, using the Haplotype caller algorithm, and variant filtration. We then used the GATK’s ASEReadCounter to count the number of reads of coastal and inland alleles present in each hybrid at each fixed SNP. Only individuals that were heterozygous for the differentiated alleles were tested for allele-specific expression and allele counts were totaled for each gene. Only genes with at least 3 heterozygous individuals were further investigated. Using the average read counts among individuals for each gene, we then calculated the log2 fold changes between coastal and inland allele expression in hybrids $\left(\log 2\left({coastal\_allele}/{inland\_allele}\right)\right)$ and compared this value to the log2 fold changes of expression between parental forms $\left(\log 2\left({coastal}/{inland}\right)\right)$. Finally, we tested for differential allele-specific expression (e.g., when an allele is more highly expressed than the other only during the migratory or non-migratory state) by comparing the log2 fold change between migratory states $\left(\log 2\left(\frac{{coastal\_allele\_nonmig}}{{coastal\_allele\_mig}}/\frac{{inland\_allele\_nonmig}}{{inland\_allele\_mig}}\right)\right)$ with log2 fold changes of expression between parental forms and migratory states $\left(\log 2\left(\frac{{coastal\_nonmig}}{{coastal\_mig}}/\frac{{inland\_nonmig}}{{inland\_mig}}\right)\right)$. For this comparison, only genes with at least three individuals in each migratory state were included and genes with at least 10 reads covering them. Note, we obtained very similar results when we estimated ratios for each individual before taking averages (vs. taking average read counts among individuals for all genes; e.g., log fold changes estimated using these two approaches were strong correlated [correlation coefficient of 0.96, *p*-value < 0.0001]).

Note, several alternate methods for testing ASE exist but are not ideal for analyses including behavioral contrasts (i.e., GxE patterns) and/or unpaired designs. As a proof of principle, we modified an existing program for testing ASE to integrate the GxE study design. Specifically, we used ASEP (with default settings and adaptive resampling set to 106, Fan et al. 2020) to calculate ASE separately for each migratory state. If GxE patterns of expression derived from cis-regulatory changes, we predicted we would find contrasting patterns for ASE between the migratory states (i.e., genes would show dissimilar levels of ASE between spring versus winter samples due to cis-regulatory changes). Instead, *p*-values were strongly correlated across states (Spearman’s rank correlation, rho = 0.35, *p*-value < 0.001) and we documented more overlap between states in the identity of genes showing significant ASE than expected by chance (*p*-value < 0.001). This result conforms with patterns reported in the results section; we fail to find evidence for differences of ASE between migratory states, suggesting that trans-regulatory changes are involved in GxE expression patterns.

### Gene ontology

We tested for enrichment of gene ontology (GO) categories using g:profiler^92^, providing lists of differentially expressed or transgressively expressed genes as the query and using the rest of the genes that were expressed in that tissue as background. *P*-values were corrected using the Benjamini-Hochberg FDR method. We used REVIGO to remove redundant and overlapping GO categories, with an allowed semantic similarity measure of 0.5^93^.

### Genomic variation

We used samples from the same parental populations used for our transcriptomic analyses (Vancouver and Kamloops, British Columbia) to estimate sequence divergence (*n* = 15 individuals/population). We used adult males, capturing them with mistnets and song playback during the breeding season. Blood samples were obtained from the brachial vein and DNA extracted using a standard phenol chloroform protocol. Whole-genome resequencing libraries were prepared by Texas A&M’s Institute for Genomic Sciences using Nextera (Illumina) DNA Flex Library Prep kits. Libraries were sequenced on a NovaSeq 6000 (paired-end 150 bp reads).

We trimmed the resulting sequences with Trim Galore! v0.3.7 (--clip_R1 15 --clip_R2 15 --three_prime_clip_R1 5 --three_prime_clip_R2 5) and aligned them to reference genomes for both the coastal and inland subspecies of Swainson’s thrush using bwa 0.7.17 (mem algorithm with default settings^94^). We converted the resulting sam files to bam format with samtools 1.11^95^ and used picardtools 2.18.27 to clean, sort and add read groups to the bam files (http://broadinstitute.github.io/picard/; CleanSam, SortSam and AddOrReplaceReadGroups, respectively). NGSutilsj (https://github.com/compgen-io/ngsutilsj) was used to identify reads that mapped uniquely to both reference genomes; all subsequent analyses were limited to the former reads and their mapping position in the inland reference genome. We chose the inland reference genome because it is more contiguous and complete. Between 97 and 98% of reads remained after the former steps, for an average read depth of 18X (range 14–39X).

We used GATK best practices to call variant and invariant sites from the final bam files (Poplink et al. 2017). Specifically, we ran HaplotypeCaller algorithm in GVCF mode for each individual and scaffold separately. We gathered data for each individual using GatherVcfs and created a database for all individuals using GenomicsDBImport. We called genotypes using GenotypeGVCFs with –all-sites flag and filtered the resulting vcf file with vcftools, removing indels, sites with >0.8 missing data, depth <7 or > 100. Finally, we used pixy^96^ to estimate a series of population genetic parameters, including F_ST_ and d_XY_ (including invariant sites) between the subspecies and nucleotide diversity within each subspecies.

The former population genetic parameters were estimated for CNEs proximate to genes (10 kb up or downstream from genes) and in introns. Wuitchik et al.^97^ generated a list of 375,591 CNEs using published data for vertebrates, merging CNEs across four studies and removing those that were too short (<20 bp; github.com/tsackton/brood-parasite-genomics/blob/master/03_CNEEs/assemble_ces.txt). We used this set in our analysis, transferring coordinates from the chicken genome used by Wuitchik et al.^97^ (GRCg6a) to the inland Swainson’s thrush genome in two steps, (1) using Progressive Cactus 2.6.4^98^ to align our reference genome to the chicken and (2) using halLiftover^99^ to transfer coordinates from the chicken to the thrush. 360,853 of the 375,591 CNEEs from Wuitchik et al. were present in the thrush genome. We used bedtools intersect to limit our analysis to CNEs proximate to genes.

### Genomic composition of hybrids

We sequenced and genotyped hybrids from the present study as part of a larger project that includes data from >800 Swainson’s thrushes across their hybrid zone. Libraries for each individual were prepared following a modified protocol based on Picelli^100,101^ et al 2014 and Schumer et al 2018. Briefly, for tagmentation 2–5 nanograms of DNA was added to a mix of TDE1 Illumina Buffer, homemade buffer (20 mM Tris-Hcl, 10 mM MgCl2) and Tn5 transposase enzyme (TDE1 - Illumina) pre-charged with custom adapters and incubated at 55 °C for 5 min. One of 96 custom indices (Tn5 i7s-IDT) were added to each sample on a plate in addition to a mastermix including an Tn5 i5 indice and OneTaq HS Quick-Load 2×. The pcr reaction included denaturation at 95 °C, annealing at 55 °C and extension at 68 °C; 12 PCR cycles were used. After amplification, 10 µl of each individual reaction was pooled and purified using AMPure XP beads (1×). Library size distribution and quality was visualized on the Bioanalyzer 1000 (Agilent, Molecular Genomics Workspace, Texas A&M University) and size selected between 350–750 base pairs (bp). All birds are sequenced to low coverage (Supplementary Data 1) on a NovaSeq 6000 (paired-end 150 bp reads) and aligned to both the coastal and inland Swainson’s thrush reference genomes as described above (steps using bwa, samtools, picardtools and ngsutilsj). Final bam files are used to impute missing genotypes using STITCH^102^. Specifically, we used bcftools 1.14 to provide STITCH with an initial set of SNPs (--min-BQ 20, --min-MQ 20, QUAL > 500, --skip-variants indels). We run STITCH in blocks of 1 Mb (with buffer of 100 kb), initiating the program in the pseudoHaploid model with values of 80 and 500 for K (ancestral haplotypes) and nGen (number of generations since population was founded). We switched to the more accurate diploid model after 36 EM for computational efficiency.

We used vcftools to filter the resulting vcf, removing indels, SNPs with more than 75% missing data and minor allele frequencies less than 5%. This filtered vcf was used to characterize the genomic composition of birds. We used the ‘HIest’ package in R^103^ for this analysis. HIest summarizes hybrid genomes using both the proportion of alleles coming from each ancestral population and interclass heterozygosity. It requires allele frequencies from parental populations; these should come from SNPs that are (nearly) diagnostic of parental forms. We used data from the parental populations described under ‘Sequence divergence’ to obtain these estimates, using 8 individuals/population to identify SNPs with F_ST_ greater than 0.95 (excluding those located on the sex chromosomes and a putative inversion on scaffold one and thinning to one SNP per 10000 bases) and estimating allele frequencies at these SNPs using the remaining 7 individuals/population. We obtained estimates of ancestry and interspecific heterozygosity for all (>800) birds but present data from those used in the present study here (Fig. S1).

A note concerning the accuracy of our imputation pipeline. We assessed its accuracy using a cross-validation approach, randomly selecting four hybrids that were originally sequenced to low-coverage to be sequenced to high coverage. We used samtools to randomly subsample reads from each individual to mimic low-coverage sequencing (at 1, 2, 4 and 7× coverage) and compared genotypes called using high-coverage data (i.e., “true” genotypes) to those estimated using low-coverage data. Comparisons were made using the squared linear correlation coefficient at a minor allele cutoff of 0.5, with r^2^ values averaged across sites and samples obtain a single summary for each coverage. Fig. S2 shows these results, with accuracy starting at ~0.95 at 1× coverage and increasing to ~0.975 at 4×.

### Meta analysis

We used the Web of Science database to identify studies on the genetics of bird migration. We limited our search to the years 2015–2021 and used the following keywords: “((bird OR avian) AND (migration OR migratory) AND gene).” Ruegg et al.^104^ conducted a similar search for years before 2015. We included their genes in our list precluding the need to rerun those years. The search returned 923 articles which were filtered for relevance. We limited our analysis to studies using de novo approaches (vs. candidate genes), examining differences in gene expression (vs. for example genomic studies) and the transition between non-migratory and migratory states. We included two studies that did not examine this transition in the same species but compared migratory and non-migratory birds in a partially migratory population of European blackbirds^38^ and two subspecies of dark-eyed junco that differ in their propensity to migrate^105^ as some of the same genes may distinguish groups in these comparisons as well.

Note, Johnston^58^ conducted a similar study in Swainson’s thrushes. They compared patterns between winter and fall migration and focused on the hypothalamus and optic chiasma. Only one gene from Johnson^58^ overlapped with results from the hypothalamus in our study (GPR17). This is not more than expected by chance and likely relates to differences in study design; for example, we sampled birds within a three-hour time window, one hour after lights out and under red light conditions to ensure circadian genes were unaffected by white light. Johnson^58^ sampled birds throughout the day and analyzed data from both brain regions together. Accordingly, we limit the number of comparisons drawn between our work and Johnson^58^.

### Reporting summary

Further information on research design is available in the Nature Portfolio Reporting Summary linked to this article.

### Supplementary information

Supplementary Information

Peer Review File

Description of Additional Supplementary Files

Supplementary Data 1–6

Reporting Summary

### Source data

Source data files

## Data Availability

The raw sequence reads generated in this study have been deposited in the SRA under BioProject PRJNA960838. Source data are provided as a Source Data file. Distrbution map obtained with permission from the BirdLife International and Handbook of the Birds of the World^106^ (http://datazone.birdlife.org/species/requestdis). Images of brains obtained from the Zebra Finch Atlas (http://www.zebrafinchatlas.org/gene_display/histological-atlas). Source data are provided with this paper.

## Code availability

A github repository with basic workflows used in this manuscript can be found here: https://github.com/kdelmore/swth_rnaseq.
