# Supplementary material for: Gene regulation and speciation in a migratory divide between songbirds
Source: Nat Commun. 2024 Jan 2;15:98. doi: 10.1038/s41467-023-44352-2 (PMC10761872; doi:10.1038/s41467-023-44352-2)
Supplement: Supplementary file 1 — Supplementary Information [file 41467_2023_44352_MOESM1_ESM.pdf]

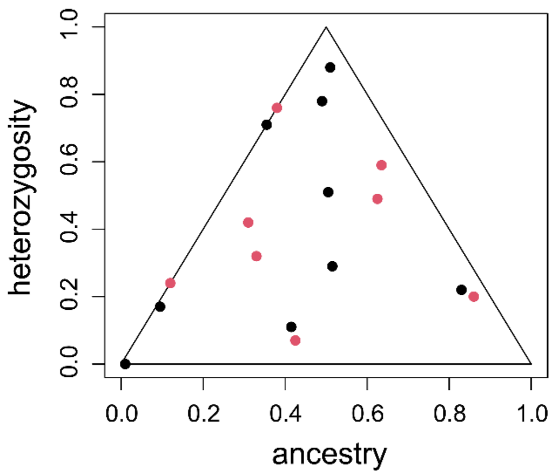

Figure S1. Hybrids included in study. Relationship between their ancestry (0=inland, 1=coastal) and interspecific heterozygosity. Birds sampled during the migratory state shown in pink.

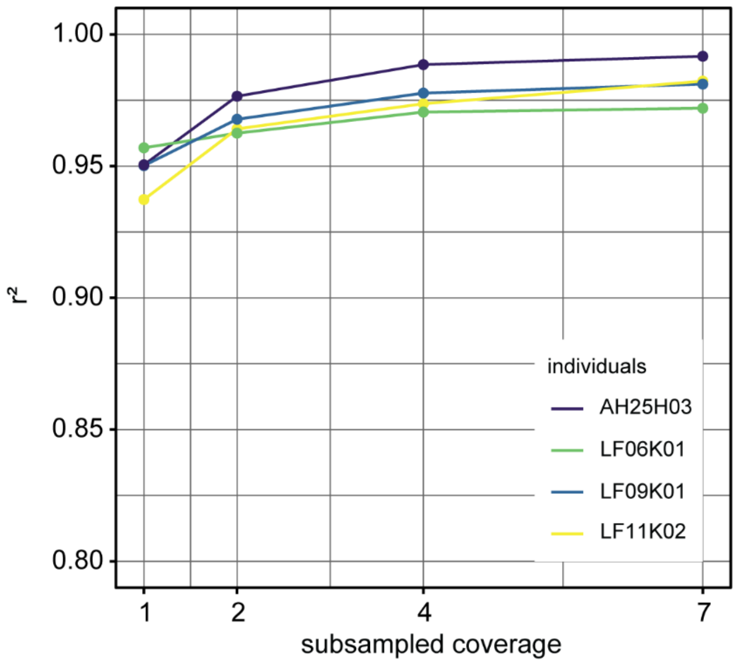

Figure S2. Imputation accuracy from STITCH. Squared correlation coefficients between imputed and actual genotypes for four hybrids sequenced to high coverage are shown.
