# Supplementary material for: Gene regulation and speciation in a migratory divide between songbirds
Source: Nat Commun. 2024 Jan 2;15:98. doi: 10.1038/s41467-023-44352-2 (PMC10761872; doi:10.1038/s41467-023-44352-2)
Supplement: Supplementary file 3 — Description of Additional Supplementary Files [file 41467_2023_44352_MOESM3_ESM.pdf]

<u>Description of Additional Supplementary Files</u>

File Name: Supplementary Data 1

Description: Individuals used in the present study, including their population of origin and when sampling was conducted. Ancestry and interspecific heterozygosity are indicated for hybrids along with the number of reads generated and mapped (using two different thresholds for masking SNPs [FST>0.1 or 0.9]).

File Name: Supplementary Data 2

Description: Results from analyses of differential expression by migratory state, including values for all five brain regions assayed and information on genes identified in previous genomic work with thrushes. Pvalues are derived from linear models run in DEseq with expression levels as the response variable, migratory state, population, and the interaction between these two variables as predictors.

File Name: Supplementary Data 3

Description: Genes from meta-analysis identifying those differentially expressed between migratory states in other species of songbird.

File Name: Supplementary Data 4

Description: Results from analyses of differential expression by subspecies and migratory state, including values for all three brain regions that included interaction genes and information on genes identified in previous genomic work with thrushes. Pvalues are derived from linear models in DEseq run with expression levels as the response variable, migratory state, population, and the interaction between these two variables as predictors.

File Name: Supplementary Data 5

Description: Results from GO analyses, including the comparison where genes were identified (GxE genes or transgressively expressed genes). FDR adjusted pvalues from a cumulative hypergeometric test run in go:profiler are shown.

File Name: Supplementary Data 6

Description: Results from linear models comparing a series of population genetic parameters at genes that were differentially expressed (or not) between the subspecies regardless of season in all five brain regions.
