# Supplementary material for: Gene regulation and speciation in a migratory divide between songbirds
Source: Nat Commun. 2024 Jan 2;15:98. doi: 10.1038/s41467-023-44352-2 (PMC10761872; doi:10.1038/s41467-023-44352-2)
Supplement: Supplementary file 5 — Reporting Summary [file 41467_2023_44352_MOESM5_ESM.pdf]

nature portfolio

Corresponding author(s): Kira Delmore
Last updated by author(s): Nov 23, 2023

Reporting Summary

Nature Portfolio wishes to improve the reproducibility of the work that we publish. This form provides structure for consistency and transparency in reporting. For further information on Nature Portfolio policies, see our Editorial Policies and the Editorial Policy Checklist.

Statistics

For all statistical analyses, confirm that the following items are present in the figure legend, table legend, main text, or Methods section.

n/a	Confirmed	
☐	☒	The exact sample size (*n*) for each experimental group/condition, given as a discrete number and unit of measurement
☐	☒	A statement on whether measurements were taken from distinct samples or whether the same sample was measured repeatedly
☐	☒	The statistical test(s) used AND whether they are one- or two-sided<br>*Only common tests should be described solely by name; describe more complex techniques in the Methods section.*
☐	☒	A description of all covariates tested
☐	☒	A description of any assumptions or corrections, such as tests of normality and adjustment for multiple comparisons
☐	☒	A full description of the statistical parameters including central tendency (e.g. means) or other basic estimates (e.g. regression coefficient) AND variation (e.g. standard deviation) or associated estimates of uncertainty (e.g. confidence intervals)
☐	☒	For null hypothesis testing, the test statistic (e.g. *F*, *t*, *r*) with confidence intervals, effect sizes, degrees of freedom and *P* value noted<br>*Give P values as exact values whenever suitable.*
☒	☐	For Bayesian analysis, information on the choice of priors and Markov chain Monte Carlo settings
☐	☒	For hierarchical and complex designs, identification of the appropriate level for tests and full reporting of outcomes
☐	☒	Estimates of effect sizes (e.g. Cohen's *d*, Pearson's *r*), indicating how they were calculated

Our web collection on statistics for biologists contains articles on many of the points above.

Software and code

Policy information about availability of computer code

Data collection	No software was used to collect data.
Data analysis	Several freely available programs were used to analyze data. Including TrimGalore! v0.3.7, vcftools 0.1.16, STAR 2.7, HTSeq-count 0.11.2, DESeq, GATK 4.2.0.0, g:profiler, REVIGO, samtools 1.11, bwa 0.7.17, picardtools 2.18.27, pixy, Hlest, progressive cactus 2.6.4, halLiftover, bcftools 1.14, STITCH. Details are available in the methods of the manuscript. And a github repository with basic workflows can be found here: https://github.com/kdelmore/swth_rnaseq/.

For manuscripts utilizing custom algorithms or software that are central to the research but not yet described in published literature, software must be made available to editors and reviewers. We strongly encourage code deposition in a community repository (e.g. GitHub). See the Nature Portfolio guidelines for submitting code & software for further information.

Data

Policy information about availability of data

All manuscripts must include a data availability statement. This statement should provide the following information, where applicable:

- Accession codes, unique identifiers, or web links for publicly available datasets
- A description of any restrictions on data availability
- For clinical datasets or third party data, please ensure that the statement adheres to our policy

Raw sequence reads generated for this project have been uploaded to the SRA under BioProject PRJNA960838 and will be made publicly available upon publication.

This link is for reviewers of the manuscript - https://dataview.ncbi.nlm.nih.gov/object/PRJNA960838?reviewer=no4c9q3phpnpfrt84dcbt878ur. Basic workflows for analysis can be found on github - https://github.com/kdelmore/swth_rnaseq.

Research involving human participants, their data, or biological material

Policy information about studies with human participants or human data. See also policy information about sex, gender (identity/presentation), and sexual orientation and race, ethnicity and racism.

Reporting on sex and gender	NA
Reporting on race, ethnicity, or other socially relevant groupings	NA
Population characteristics	NA
Recruitment	NA
Ethics oversight	NA

Note that full information on the approval of the study protocol must also be provided in the manuscript.

Field-specific reporting

Please select the one below that is the best fit for your research. If you are not sure, read the appropriate sections before making your selection.

☐ Life sciences ☐ Behavioural & social sciences ☒ Ecological, evolutionary & environmental sciences

For a reference copy of the document with all sections, see nature.com/documents/nr-reporting-summary-flat.pdf

Ecological, evolutionary & environmental sciences study design

All studies must disclose on these points even when the disclosure is negative.

Study description	We quantified differential expression between two subspecies of Swainson's thrushes and their hybrids during both the non-migratory and migratory period. 36 birds were included in the study. 18 hybrids (9 from each migratory state); 9 coastals (4 from the non migratory state and 5 from the migratory state); and 9 inlands (4 from the non migratory state and 5 from the migratory state).
Research sample	We focused on two subspecies of Swainson's thrushes and their hybrids in the present study. We euthanized five birds/parental subspecies (coastal and inland) during the non-migratory season and five birds/parental subspecies during the spring migratory season. Twice as many hybrids were euthanized in each season (8 during the non-migratory period and 10 during the non-migratory period). Separate libraries were constructed for each tissue and individual. All birds were captured as juveniles in British Columbia during fall migration (Aug). All birds were males.
Sampling strategy	Samples sizes of 3-6 are considered sufficient for analyses of differential expression.
Data collection	KED recorded data during capture, dissection and RNA extractions, including the individual IDs, ancestry, behavioral conditions (non-migratory vs. non-migratory season) and brain regions. Data were originally recorded with pen and paper before being input into excel sheets.
Timing and spatial scale	Birds were captured in August 2020 and 2021 when they had left the nest and were independent. Capture occurred in populations adjacent to the hybrid zone and directly at its center. Non-migratory birds were euthanized between January 20 and February 2 and migratory birds between May 28 and April 15. These are the dates when birds were exhibiting appropriate behavior, as documented using motion detectors (diurnal for non-migratory and nocturnal for migratory). Tissue was collected at night, 1-hour following the onset of darkness in the subject room and over no more than three hours (3 - 4 individuals processed/day) to ensure gene expression related to migration was initiated but birds were collected around the same time. Tissue collection was performed under red light conditions to avoid artificial changes in gene expression.
Data exclusions	No data were excluded.
Reproducibility	Each protocol employed in this study was well thought out and practiced beforehand. Care was taken to ensure all protocols are well described in the manuscript. As a result, all results should be reproducible.
Randomization	Birds were captured using mistnets and playback. There was no pattern to capture except that hybrids were selected based on ancestry calls from RFLPs. We chose birds exhibiting the highest levels of ancestry as these birds provide the most power for allele-specific expression analyses. Birds had to exhibit the correct behavior for at least 10 days prior to euthanization. Birds fitting this criteria were identified each day and randomly chosen for sacrifice. RNA was extracted in a random order and sequencing was randomized across lanes.
Blinding	Birds are given unique identifiers that have nothing to do with their ancestry of behavioral condition. Unless necessary (e.g., when

Blinding	conducting analyses) these features would not have been available to those conducting protocols (e.g., ancestry would not have been known during euthanization).

Did the study involve field work? ☒ Yes ☐ No

Field work, collection and transport

Field conditions	Late summer on the breeding grounds for Swainson's thrushes.
Location	Vancouver (-123.2115, 49.2480), Pemberton (50.26474,-122.867) and Kamloops (50.9039, -120.3131) in British Columbia, Canada.
Access & import/export	All experiments were performed in accordance with relevant guidelines and regulations. Protocols were approved by the Institutional Animal Care and Use Committee at Texas A&M (IACUC 2019-0066) and permits were obtained from Environment and Climate Change Canada (SC-BC-2020-0016), the U.S. Fish and Wildlife Service (MB49986D-0) and Texas Parks and Wildlife Commission (SPR-0419-067).
Disturbance	Minimal disturbance (e.g., existing paths used instead of creating our own net lanes).

Reporting for specific materials, systems and methods

We require information from authors about some types of materials, experimental systems and methods used in many studies. Here, indicate whether each material, system or method listed is relevant to your study. If you are not sure if a list item applies to your research, read the appropriate section before selecting a response.

Materials & experimental systems

n/a	Involved in the study
☒	☐ Antibodies
☒	☐ Eukaryotic cell lines
☒	☐ Palaeontology and archaeology
☐	☒ Animals and other organisms
☒	☐ Clinical data
☒	☐ Dual use research of concern
☒	☐ Plants

Methods

n/a	Involved in the study
☒	☐ ChIP-seq
☒	☐ Flow cytometry
☒	☐ MRI-based neuroimaging

Animals and other research organisms

Policy information about studies involving animals; ARRIVE guidelines recommended for reporting animal research, and Sex and Gender in Research

Laboratory animals	No lab animals were used in the study.
Wild animals	Swainson's thrushes. 9 coastal thrushes; 9 inland thrushes and 18 hybrid thrushes. Juveniles and male. Captured using mistnets and playback. Transported in cages via car and airplane. Euthanized via decapitation. All following standard ornithological protocols and permits obtained for the work.
Reporting on sex	All males. First step in large project. Females will be next.
Field-collected samples	All birds were relocated to an animal facility at Texas A&M University where they were housed in individual cages and fed a diet consisting of berries, mealworms, egg, crackers, cottage cheese and red meat ad libitum84. We used previous data collected from free-flying hybrids fitted with geolocators to adjust the photoperiod experienced by captive birds to mimic natural conditions23,24. Specifically, we gradually decreased the photoperiod from the migratory state of 25 degrees Celsius and 14 hours of light (L):10 hours of dark (D) to the non-migratory state of 22 degrees Celsius and 11L:13D in September– October. Birds were held in this non-migratory state until February-March when the photoperiod was gradually increased to 16L:8D, mimicking spring migration and eventual arrival on the breeding grounds.
Ethics oversight	All experiments were performed in accordance with relevant guidelines and regulations. Protocols were approved by the Institutional Animal Care and Use Committee at Texas A&M (IACUC 2019-0066) and permits were obtained from Environment and Climate Change Canada (10921; SC-BC-2019-0016; SC-BC-2020-0016), the U.S. Fish and Wildlife Service (MB49986D-0; LE51239D-0), Texas Parks and Wildlife Commission (SPR-0419-067) and the United States Department of Agriculture (RIV19148289; RIV20152472).

Note that full information on the approval of the study protocol must also be provided in the manuscript.
