# Supplementary material for: Gene regulation and speciation in a migratory divide between songbirds
Source: Nat Commun. 2024 Jan 2;15:98. doi: 10.1038/s41467-023-44352-2 (PMC10761872; doi:10.1038/s41467-023-44352-2)
Supplement: Supplementary file 2 — Peer Review File [file 41467_2023_44352_MOESM2_ESM.pdf]

nature portfolio

Peer Review File

Gene regulation and speciation in a migratory divide between songbirds

Open Access This file is licensed under a Creative Commons Attribution 4.0 International License, which permits use, sharing, adaptation, distribution and reproduction in any medium or format, as long as you give appropriate credit to the original author(s) and the source, provide a link to the Creative Commons license, and indicate if changes were made. In the cases where the authors are anonymous, such as is the case for the reports of anonymous peer reviewers, author attribution should be to 'Anonymous Referee' followed by a clear attribution to the source work. The images or other third party material in this file are included in the article's Creative Commons license, unless indicated otherwise in a credit line to the material. If material is not included in the article's Creative Commons license and your intended use is not permitted by statutory regulation or exceeds the permitted use, you will need to obtain permission directly from the copyright holder. To view a copy of this license, visit http://creativecommons.org/licenses/by/4.0/.

REVIEWER COMMENTS

Reviewer #1 (Remarks to the Author):

The authors present an analysis of gene expression in five brain regions during migratory and non-migratory states, from two subspecies of Swainson's thrush and their hybrids. This is an interesting investigation of the molecular basis underlying behavioral variation that likely plays an important role in postzygotic reproductive isolation. Much less is known about the genetics underlying behavioral postzygotic reproductive isolating barriers versus postzygotic barriers such as sterility or inviability. This is therefore an important and interesting contribution to the field of the genomics of speciation. Additionally, the manuscript is very clearly written.

With this in mind, one of my main suggestions is that the introduction is re-organized and re-framed to focus more on how this work adds to our knowledge about speciation and reproductive isolation, versus how it adds to our general knowledge about behavioral genetics. The first two paragraphs of the introduction are very focused on methods, which is less interesting than the general questions these methods can address. Moving much of the context provided in the third paragraph of the introduction to the beginning would help greatly. The commentary about gaps in understanding of behavioral genetics provided in the current first and second paragraphs could still be included, but should not be the main focus. This re-framing would actually work well with much of how the abstract and the discussion are already presented.

From what I understand, the birds were captured prior to their fall migration period, which they all then experienced in captivity, under common conditions. The first sampling period was during the winter non-migratory period, with the migratory sampling done during the spring season (when they would be migrating to their breeding sites). Is it possible that missing that fall migration could have affected gene expression during both the winter and the spring? Also, why was the fall migration period not sampled? Would the authors expect there might be differential gene expression between the two different migration periods? And could any of those differences have potentially interacted with the subspecies/hybrid designation? I would like this aspect of the study design/sampling to be explained, and the potential for differences across migration seasons to be discussed.

Minor comments:

1. Lines 36-37: revise to read "...between the roles of cis- versus trans-regulatory changes in generating behavioral variation."

2. Line 78: revise to read "...differential expression and to examine patterns..."

3. Line 226: delete "with" and change "exhibiting" to "exhibited"

4. Section on Tissue collection (starting at line 420): Please include details on how the birds were euthanized early in this section. It is mentioned later that they were decapitated, but this is an important detail that needs to be addressed directly and early.

5. Line 427: Should May be March?

6. Lines 480-481: Is an FDR-adjusted p-value <0.10 standard for gene expression studies? I would have expected significance to be assigned to an adjusted p-value of <0.05 by convention.

7. Line 894 (Figure 5 legend): (d) should be (c)

8. Line 896 (Figure 5 legend): insert "one" between "and" and "that"

Reviewer #2 (Remarks to the Author):

In this study, Louder et al explore gene expression in differentially migrating populations of the Swainson’s thrush and in their hybrids. Bird migration is one of the most well-known phenomena in Nature, but despite its strong genetic basis in many species, the genetic architecture of migratory behavior remains poorly understood. Migratory behavior is also thought to be important in speciation. This is the case in the Swainson’s thrush, where migratory behavior in hybrids is different from the parental populations and likely to lead to reduced fitness. Addressing both the genetics underlying an understudied complex trait such migratory behavior and its effect on speciation is of broad interest to biologists.

Overall, the paper is clearly written and most of the results are presented well. The figures are also generally nice and not overly complicated. In my opinion, the strongest asset of this study is the quality of the expression data. Previous expression studies on migration have for example used whole-brain to find the genetic basis of migration, which is easier to obtain, but may lack the necessary resolution to find meaningful behavior-related differences. It is also difficult to obtain good migratory phenotypes. For example, many songbirds migrate at night, which makes it difficult to obtain individuals from wild populations that truly express migratory behavior. Here, Louder et al have used very controlled settings and also investigated several specific regions of the brain that previously have been connected with migratory behavior. Another important aspect of this study is that they have included data from hybrids. This allows for the study of genes that may underlie the different migratory behavior in hybrids and that could lead to reduced fitness.

When it comes to the results, I think the weakest link in this study is the allele-specific expression analysis and how it is used to infer the relative importance of cis- and transregulatory elements. There is a correlation between allele-specific expression in hybrids and parental expression when not considering migratory state, but a lack of correlation when testing allele-specific expression when taking into

consideration both subspecies and migratory state. The authors interpret this as evidence that changes in the latter category are mainly driven by transregulatory mechanisms.

To begin with, I am not entirely sure how the allele-specific expression has been quantified. They mention that they only included samples heterozygous for subspecies-specific alleles, but exactly how they were merged together for a gene and across individuals could be described in a clearer way. Were the different alleles summed across the different samples for the same gene? If that is the case, I don't think that it is an optimal approach.

When it comes to allele-specific expression, it is easy to see how it can be quantified and interpreted when looking within specific heterozygous individuals. The environment within a single individual serves as a control, where differences between the alleles in expression (assuming they can be measured properly) should be caused by regulatory differences. However, contrasting allele counts between different individuals does not seem as intuitive to me. Judging from the equation on line 177, this must have been the case when contrasting coastal and inland alleles between migratory and non-migratory hybrids. Maybe I have not fully understood the equation, but again, going from individual allele-specific counts for specific sites into some gene-based summary for a group of individuals needs to be better described. In any case, this measurement is more complicated than the one for the hybrids overall stated on line 496 and involves several ratios. I am not surprised if there would be more variance when comparing the alleles across hybrid groups in this way and hence a weaker correlation with the measurements in the parental populations.

Related to this, what are the hybrid indices in the different groups? Are they more or less the same? The number of hybrid genotypes in the different groups could potentially influence the contrast between the groups. It is mentioned that there should have been at least three heterozygous samples when checking for allele-specific expression. Was this also true when comparing the migrating – non-migrating hybrids? That is, was it necessary for both of the groups to have at least three samples?

Furthermore, I cannot find any information how many genes remained for checking allele-specific expression. Overall, there is no detailed information on how much RNA was sequenced in the end for different samples and tissues. This information should be added in a supplementary table.

When it comes to quantifying ASE among different individuals or groups, there seem to be better or at least more intuitive methods. For example, Mugal et al 2020 (Genome Research. 30(12):1727-1739) and van Beek et al 2023 (Sci Rep 13. 564) have first calculated ratios within samples and then used other methods to summarize within groups or contrast between populations. Another method that could potentially be used is ASEP (Fan et al, https://doi.org/10.1371/journal.pgen.1008786). To explore the GxE effect, I also wonder if it would be clearer to directly compare ASE in only the migratory hybrids with differences between migratory samples of the two populations.

In addition, the authors also find that there is elevated FST between the constitutively differentially expressed genes between the subspecies. While there may be a biological reason for this, allele-specific expression is known to be sensitive to reference bias. They have already masked the genome for nearly fixed SNPs between the populations, which I think is great. However, I still wonder if there may be residual reference bias that could explain this pattern in FST. FST>0.9 seems to be quite conservative threshold for masking and it would be interesting to see if this pattern still holds when lowering the threshold. What happens for example when lowering to FST>0.2 or something similar, or even when masking for all of the variants? An alternative method to try would be the WASP pipeline (https://github.com/bmvdgeijn/WASP).

Also, I do not feel very convinced that there necessarily should be a correlation between overall gene differentiation (and other diversity measurements) and allele-specific expression. For small genes, a selection signal in the regulatory elements, for example in the promoter region, may also cover a substantial part of the gene. For larger genes, however, this signal could easily be missed. That is, the absence of this overall gene pattern does not mean that there cannot be cis-regulatory changes. To get a better resolution, one could specifically explore conserved elements upstream of the gene or in introns, which has been done in Mugal et al 2020 (Genome Research. 30(12):1727-1739).

Another major concern I have is the estimation of hybrid indices. To calculate these estimates, the authors have used low coverage whole-genome sequencing in combination with the software Stich to impute missing genotypes. Since there are good reference samples from each of the populations included in this study, it would be good to check if a reference panel-based imputation software would give a similar result. Even with the best imputation methods for this data set, I imagine that there can be some issues with estimating hybrid proportions and heterozygosity when coverage is very low. It would therefore be interesting to see how well the results in Figure S1, and in particular heterozygosity, correlate with the mean coverage of the samples. Ideally, the hybrid information and the coverage information

of each sample should be added as extra columns to Table S1.

It is a bit unfortunate that the hybrids have not been sequenced to a higher coverage, as this would been beneficial for the allele-specific expression. In this case, it would have been easier to detect which samples were heterozygous for the fixed differences, which would likely have given more power to the analyses.

Minor comments

Lines 150-151: Delmore references should be Delmore et al, right?

Line 467: Provide information about the annotations of the assembly. How was the annotation created, what release is it and how many genes in total?

Line 522: Be more specific about the cleaning in picardtools. Remove duplicates and/or some other command?

Line 834: It would have been good to add the detailed workflows for the reviewing process, as it would have made it clearer how the expression analyses actually had been performed.

Table S4: It says in the table header that there are five brain regions, but there only seems to be data for three regions

Reviewer #1 (Remarks to the Author):

The authors present an analysis of gene expression in five brain regions during migratory and non-migratory states, from two subspecies of Swainson's thrush and their hybrids. This is an interesting investigation of the molecular basis underlying behavioral variation that likely plays an important role in postzygotic reproductive isolation. Much less is known about the genetics underlying behavioral postzygotic reproductive isolating barriers versus postzygotic barriers such as sterility or inviability. This is therefore an important and interesting contribution to the field of the genomics of speciation. Additionally, the manuscript is very clearly written.

With this in mind, one of my main suggestions is that the introduction is re-organized and re-framed to focus more on how this work adds to our knowledge about speciation and reproductive isolation, versus how it adds to our general knowledge about behavioral genetics. The first two paragraphs of the introduction are very focused on methods, which is less interesting than the general questions these methods can address. Moving much of the context provided in the third paragraph of the introduction to the beginning would help greatly. The commentary about gaps in understanding of behavioral genetics provided in the current first and second paragraphs could still be included, but should not be the main focus. This re-framing would actually work well with much of how the abstract and the discussion are already presented.

We appreciate this comment but have chosen to keep much of our introduction as is. We have two main reasons for this decision:

1. **The current organization matches how the rest of the manuscript is organized. Specifically, the first two paragraphs describe gaps in our knowledge of behavioral genetics and the third paragraph describes gaps in our knowledge of speciation genetics. This is exactly how we present our results and discussion (starting with differences between migratory states [behavioral genetics] before moving on to differences related to speciation). We could reorganize the rest of the manuscript as well but think that would affect the logic of our results, where we start by describing differences between states before moving on to those at the intersection between these states and the subspecies (i.e., those we think are most relevant for speciation).**

2. **We think many readers will be interested in how our approach overcomes current methodological limitations in the field of behavioral genetics. These limitations have been the focus of several recent high-profile reviews (e.g., Bengston et al. 2018 Nat Ecol Evol; Niepoth and Bendesky 2020 Annu Rev Genomics Hum Genet) and some of our results (e.g., our finding that trans-regulatory differences are more important for subspecies-differences in gene regulation) counter prevailing views in the field. We think these findings relate to improvements in our approach to studying these topics and should be highlighted. Reviewer #2 also highlighted our approach as one of "strongest assets" of our study.**

From what I understand, the birds were captured prior to their fall migration period, which they all then experienced in captivity, under common conditions. The first sampling period was during the winter non-migratory period, with the migratory sampling done during the spring

season (when they would be migrating to their breeding sites). Is it possible that missing that fall migration could have affected gene expression during both the winter and the spring? Also, why was the fall migration period not sampled? Would the authors expect there might be differential gene expression between the two different migration periods? And could any of those differences have potentially interacted with the subspecies/hybrid designation? I would like this aspect of the study design/sampling to be explained, and the potential for differences across migration seasons to be discussed.

Thank you for this comment. We have added discussion of fall migration to the revised manuscript. Specifically, we note that future work assaying gene expression on fall migration would be valuable for determining if signals differ between the two legs of migration (lines 288-295). We expect some signatures to stay the same (e.g., those related to the circadian clock which likely helps with the transition between diurnal and nocturnal behavior on both legs of migration) but others to differ (e.g., we know wild thrushes take different routes on fall and spring migration independent of ancestry and differences in gene expression may contribute to this variation, Delmore et al. 2012 Proc B).

Concerning why we didn't include fall migration, it was simply too logistically challenging. We wanted to use juvenile thrushes who had not migrated before, and it is difficult to find large numbers of juveniles before the end of the breeding season at the center of the hybrid zone where densities are lower. Accordingly, the birds would only have had 1-2 weeks in captivity before we would have started the experiment and we did not think that was enough time to ensure the signatures we were getting were related to migration vs. stress associated with captivity.

And note, we do not think our birds missed fall migration. Fall migration might have been disturbed in the time it took us to bring birds into the vivarium (1 day in late Aug) but once they were in the vivarium, they continued experiencing the same conditions they would have if they were in the wild (long days transitioning to short days and cooler temperatures for the winter). We did not start monitoring nocturnal activity with motion sensors until October but know from infrared cameras that birds were exhibiting the expected nocturnal behavior in September.

Apologies but we are not entirely sure how to interpret this comment: "And could any of those differences have potentially interacted with the subspecies/hybrid designation?" **We did not use differences in gene expression to designate birds as parental or hybrid. Parental birds came from different geographic sites than hybrids (Vancouver [coastal] and Kamloops [inland] vs. Pemberton).**

Minor comments:
1. Lines 36-37: revise to read "...between the roles of cis- versus trans-regulatory changes in generating behavioral variation."

Change made (lines 37-38).

2. Line 78: revise to read "...differential expression and to examine patterns..."

Change made (line 79).

3. Line 226: delete "with" and change "exhibiting" to "exhibited"

Change made (line 232).

4. Section on Tissue collection (starting at line 420): Please include details on how the birds were euthanized early in this section. It is mentioned later that they were decapitated, but this is an important detail that needs to be addressed directly and early.

Change made (lines 437-438).

5. Line 427: Should May be March?

Thank you, change made (lines 442).

6. Lines 480-481: Is an FDR-adjusted p-value <0.10 standard for gene expression studies? I would have expected significance to be assigned to an adjusted p-value of <0.05 by convention.

Yes, an FDR-adjusted p-value of 0.10 is commonly used in RNA-seq studies and is the default threshold in the DESeq analysis program -- https://bioconductor.org/packages/release/bioc/vignettes/DESeq2/inst/doc/DESeq2.pdf

7. Line 894 (Figure 5 legend): (d) should be (c)

Change made (line 975).

8. Line 896 (Figure 5 legend): insert "one" between "and" and "that"

Change made (line 977).

Reviewer #2 (Remarks to the Author):

In this study, Louder et al explore gene expression in differentially migrating populations of the Swainson's thrush and in their hybrids. Bird migration is one of the most well-known phenomena in Nature, but despite its strong genetic basis in many species, the genetic architecture of migratory behavior remains poorly understood. Migratory behavior is also thought to be important in speciation. This is the case in the Swainson's thrush, where migratory behavior in hybrids is different from the parental populations and likely to lead to reduced fitness. Addressing both the genetics underlying an understudied complex trait such migratory behavior and its effect on speciation is of broad interest to biologists.

Overall, the paper is clearly written and most of the results are presented well. The figures are also generally nice and not overly complicated. In my opinion, the strongest asset of this study is the quality of the expression data. Previous expression studies on migration have for example used whole-brain to find the genetic basis of migration, which is easier to obtain, but may lack the necessary resolution to find meaningful behavior-related differences. It is also difficult to obtain good migratory phenotypes. For example, many songbirds migrate at night, which makes it difficult to obtain individuals from wild populations that truly express migratory behavior. Here, Louder et al have used very controlled settings and also investigated several specific regions of the brain that previously have been connected with migratory behavior. Another important aspect of this study is that they have included data from hybrids. This allows for the study of genes that may underlie the different migratory behavior in hybrids and that could lead to reduced fitness.

When it comes to the results, I think the weakest link in this study is the allele-specific expression analysis and how it is used to infer the relative importance of cis- and transregulatory elements. There is a correlation between allele-specific expression in hybrids and parental expression when not considering migratory state, but a lack of correlation when testing allele-specific expression when taking into consideration both subspecies and migratory state. The authors interpret this as evidence that changes in the latter category are mainly driven by transregulatory mechanisms.

Thank you. Your feedback helped improve this element of our manuscript. Please see our responses to your individual comments below. To summarize, we have added clarity to the manuscript, conducted one of the analyses you suggested and would like to highlight that we use two independent approaches to distinguish between cis- and transregulation – not only ASE but also genomic differentiation. Both approaches support transregulation for GxE. As does our observation that there is no overlap between previous genomic approaches (scans for selection and GWAS) and GxE expression patterns reported here.

To begin with, I am not entirely sure how the allele-specific expression has been quantified. They mention that they only included samples heterozygous for subspecies-specific alleles, but exactly how they were merged together for a gene and across individuals could be described in a clearer way. Were the different alleles summed across the different samples for the same gene? If that is the case, I don't think that it is an optimal approach.

Thank you for this comment. This was unclear on our part. We did not sum across samples – we counted reads within an individual at each allele and summed per gene, then used the averaged across individuals to identify the allele ratio (if meeting the 3 individual threshold). We have added clarity to the text (lines 513-515).

When it comes to allele-specific expression, it is easy to see how it can be quantified and interpreted when looking within specific heterozygous individuals. The environment within a single individual serves as a control, where differences between the alleles in expression (assuming they can be measured properly) should be caused by regulatory differences. However, contrasting allele counts between different individuals does not seem as intuitive to me. Judging from the equation on line 177, this must have been the case when contrasting coastal and inland alleles between migratory and non-migratory hybrids. Maybe I have not fully understood the equation, but again, going from individual allele-specific counts for specific sites into some gene-based summary for a group of individuals needs to be better described. In any case, this measurement is more complicated than the one for the hybrids overall stated on line 496 and involves several ratios. I am not surprised if there would be more variance when comparing the alleles across hybrid groups in this way and hence a weaker correlation with the measurements in the parental populations.

Hopefully we clarified how ASE was estimated in our response to the previous comment.

Concerning whether adding the additional term in the GxE equation introduced variation and made it harder to find a correlation, we do not think that was the case. First, variation is not greater in the ratios estimated for GxE vs. just between subspecies. That can be seen in Figure 4a and we have run a statistical comparison to support this qualitative observation (var.test in R all p-values > 0.1). Second, at least two other comparable studies (York et al. 2018 PNAS; Hu et al. 2022 Cell Report) used the same set of equations and did find GxE effects for ASE ratios between species and conditions. Accordingly, it is possible to uncover GxE effects using this equation.

Related to this, what are the hybrid indices in the different groups? Are they more or less the same? The number of hybrid genotypes in the different groups could potentially influence the contrast between the groups. It is mentioned that there should have been at least three heterozygous samples when checking for allele-specific expression. Was this also true when comparing the migrating – non-migrating hybrids? That is, was it necessary for both of the groups to have at least three samples?

Concerning hybrid indices, there is no significant difference between the groups (t.test for ancestry t=-0.40, p=0.69; t.test for heterozygosity t=0.16 p=0.87).

Concerning if there needed to be at least three heterozygous samples when testing for ASE when comparing between states, yes – both groups were required to have at least three samples for any comparisons of ASE. Furthermore, for comparisons between states, we only compared the allele ratios for a given gene if it met the three-sample threshold in each group. We included a statement to clarify in lines 522-523.

Furthermore, I cannot find any information how many genes remained for checking allele-specific expression. Overall, there is no detailed information on how much RNA was sequenced in the end for different samples and tissues. This information should be added in a supplementary table.

Thank you. This information should have been included in our original manuscript. We have added the number of genes involved in ASE comparisons to the results (lines 186 -190) and coverage to the Methods (lines 479-480).

When it comes to quantifying ASE among different individuals or groups, there seem to be better or at least more intuitive methods. For example, Mugal et al 2020 (Genome Research. 30(12):1727-1739) and van Beek et al 2023 (Sci Rep 13. 564) have first calculated ratios within samples and then used other methods to summarize within groups or contrast between populations. Another method that could potentially be used is ASEP (Fan et al, https://doi.org/10.1371/journal.pgen.1008786). To explore the GxE effect, I also wonder if it would be clearer to directly compare ASE in only the migratory hybrids with differences between migratory samples of the two populations.

Thank you for these suggestions. Unfortunately some of these methods are not applicable to our study. For example, RPASE used by Mugal et al. (2020) only tests for ASE at the level of the individual (testing for ASE at every locus within each individual). We are interested in comparing across groups (with or without a behavioral contrast). As the reviewer notes, there are ways to extend RPASE to the group-level but these modifications are not direct and cannot extend to behavioral contrasts (i.e., test for GxE effects). We are interested in ASE at both the level of subspecies and subspecies x migratory state.

We were able to modify methods described by Fan et al. 2020 to our study. These authors developed the program ASEP, which incorporates a mixture model with a subject-specific random effect to identify ASE within a gene between individuals. It also accounts for multi-SNP correlations within the same gene. There is the option in ASEP to compare between conditions (i.e., to test for ASE at GxE genes) but this is only for paired samples within the same individual (e.g., pre- vs post-treatment). Our samples are not paired. Accordingly, we modified their approach by separately testing for ASE in each migratory state. We expected to find contrasting ASE patterns between migratory and non-migratory states if differences in cis-regulatory regions drive differences in gene expression/migratory behavior between both the subspecies. In other words, if a cis-regulatory region influenced the differences between migratory states, then we expected to find stronger ASE patterns in one season versus the other. Instead, p-values were strongly correlated across states (Spearman's rank correlation, rho = 0.35, p-value < 0.001) and we documented more overlap between states in the identity of genes showing significant ASE than expected by chance (p-value < 0.001). This result is in line with our original findings, providing further evidence that differences in gene expression/behavior between migratory states in thrushes are largely driven by divergent trans-(vs. cis) regulatory regions.

We have added the former analysis with ASEP to our revised manuscript (line 193; lines 525-537) but have chosen to keep our original analyses in the main text as (1) it is a more direct test of ASE with a behavioral contrast and (2) it is an accepted/published method for comparing ASE with and without a behavioral context (York et al. 2018 PNAS; Hu et al. 2022 Cell Reports). That was not the original formulation for Fan et al. 2020.

Concerning the reviewer's suggestion that we compare allele ratios in each migratory state directly, we do not think this approach would provide a more direct estimate of the GxE effect. Without the non-migratory state included, this test would be essentially equivalent to the between subspecies ASE comparison that we already have; the only way to directly test for GxE effects is to include both migratory and non-migratory states in the same analysis. As noted already, the same approach has been used by other authors in the literature (York et al. 2018 PNAS; Hu et al. 2022 Cell Reports).

We are open to further discussion on this topic but would like to finish by highlighting the fact that we included a complementary analysis in our original manuscript as well. This analysis uses an entirely different dataset and approach to distinguish between cis- and trans-regulation (whole genome resequencing data and estimates of genomic variation). And supports results from our analysis of ASE, suggesting GxE expression derives from changes in trans-regulatory region. We have highlighted these results in the Abstract (line 20) and they are also in line with our observation that there is limited overlap between patterns of gene expression and previous genomic studies in thrushes (selection scans, [Delmore et al. 2015] and GWAS for migration [Delmore et al. 2016]; highlighted between lines 314 and 317). Combined, all of these analyses and observations provide strong support for trans-regulatory changes underlying GxE patterns in thrushes.

In addition, the authors also find that there is elevated FST between the constitutively differentially expressed genes between the subspecies. While there may be a biological reason for this, allele-specific expression is known to be sensitive to reference bias. They have already masked the genome for nearly fixed SNPs between the populations, which I think is great. However, I still wonder if there may be residual reference bias that could explain this pattern in FST. FST>0.9 seems to be quite conservative threshold for masking and it would be interesting to see if this pattern still holds when lowering the threshold. What happens for example when lowering to FST>0.2 or something similar, or even when masking for all of the variants? An alternative method to try would be the WASP pipeline (https://github.com/bmvdgeijn/WASP).

Thank you for this comment, it likely derives from a lack of clarity on our part. Specifically, we used an entirely different set of data and bioinformatics pipeline when estimating FST. We used high coverage WGS (vs. RNAseq) data from parental populations. We have reference genomes for both subspecies of thrushes. We aligned these data to both references and limited subsequent analyses to reads that aligned uniquely to both. We used mapping positions from the inland reference as it is more complete, although both references are of high quality (e.g., 96.8% and 94.2% of avian orthologs are present in the inland and coastal references, respectively). This approach should remove all reference bias from our dataset. We have added clarity to our methods between lines 555-566. No masking was done so we do not have the need to test different FST thresholds.

Also, I do not feel very convinced that there necessarily should be a correlation between overall gene differentiation (and other diversity measurements) and allele-specific expression. For small genes, a selection signal in the regulatory elements, for example in the promoter region, may also cover a substantial part of the gene. For larger genes, however, this signal could easily be missed. That is, the absence of this overall gene pattern does not mean that there cannot be cis-regulatory changes. To get a better resolution, one could specifically explore conserved elements upstream of the gene or in introns, which has been done in Mugal et al 2020 (Genome Research. 30(12):1727-1739).

We have followed the reviewer's suggestion here, running a series of new analyses to identify CNEs in our system and using the same approach described in Mugal et al. 2020 to compare genomic variation at CNEs up and downstream of genes and in their introns. Additional methods can be found between lines 577-586 in the revised manuscript but briefly, Wuitchik et al. (bioRxiv, https://doi.org/10.1101/2022.05.22.492970) built a set of CNEs using published data from vertebrates. Coordinates for these CNEs corresponded to the chicken. Accordingly, we used Progressive Cactus to align our reference genome to the chicken and halLiftover to convert coordinates from the chicken genome to our genome. We identified CNEs up and downstream of genes in the Swainson's thrushe genome and in introns and re-estimated FST, dXY and nucleotide diversity in these regions. Our results remain unchanged – we still find an association between genomic variation at genes DE between the pure forms but not at genes that exhibit GxE patterns (lines 219-226).

Another major concern I have is the estimation of hybrid indices. To calculate these estimates, the authors have used low coverage whole-genome sequencing in combination with the software Stich to impute missing genotypes. Since there are good reference samples from each of the populations included in this study, it would be good to check if a reference panel-based imputation software would give a similar result. Even with the best imputation methods for this data set, I imagine that there can be some issues with estimating hybrid proportions and heterozygosity when coverage is very low. It would therefore be interesting to see how well the results in Figure S1, and in particular heterozygosity, correlate with the mean coverage of the samples. Ideally, the hybrid information and the coverage information of each sample should be added as extra columns to Table S1.

We are confident our imputation pipeline provided accurate genotype estimates for our hybrids.

First, we assessed the accuracy of our pipeline using a cross-validation approach, randomly selecting four hybrids that were originally sequenced to low-coverage to be sequenced to high coverage. We used samtools to randomly subsample reads from each individual to mimic low-coverage sequencing (at 1, 2, 4 and 7x coverage) and compared genotypes called using high coverage data (i.e., "true" genotypes) to those estimated using low-coverage data. Comparisons using the squared linear correlation coefficient at a minor allele cutoff of 0.5, with r2 values averaged across sites and samples obtain a single summary for each coverage. Figure S2 shows these results, with accuracy starting at ~0.95 at 1x coverage and

increasing to ~0.975 at 4x. And details on this analysis can be found between lines 625 and 633 in the revised manuscript.

Concerning the use of a reference panel, that was our original approach when imputing missing genotypes with low coverage data. We used loimpute and followed methods described in Fuller et al. 2020 Science for this analysis. We assessed imputation accuracy following the methods described above (subsampling the same hybrid individuals to low coverage) and it was lower with this reference panel-based approach. The plot below shows data for one hybrid (LF06K01) and can be compared with results for the same birds in Figure S2 where imputation accuracy plateaued at 0.97 and 2X.

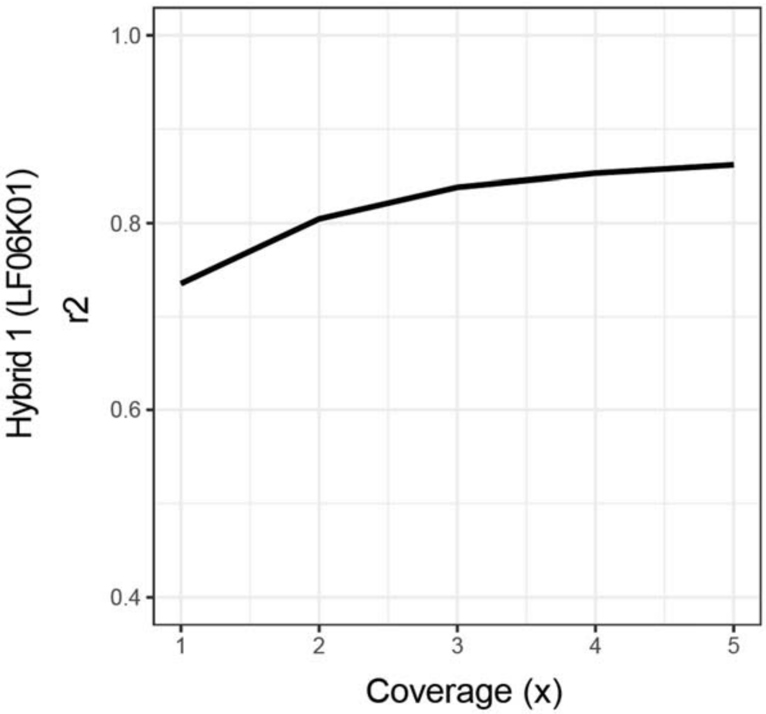

Concerning whether there is an association between coverage and heterozygosity, no – that is not the case (linear model coverage ~ heterozygosity; R2 = 0.0011, p = 0.90).

Finally, as requested, we have added hybrid and coverage information to Table S1.

It is a bit unfortunate that the hybrids have not been sequenced to a higher coverage, as this would been beneficial for the allele-specific expression. In this case, it would have been easier to detect which samples were heterozygous for the fixed differences, which would likely have given more power to the analyses.

We agree that it would have been interesting to use higher coverage genomic sequencing to identify heterozygous samples. However, for ASE tests, we would only be able to investigate the heterozygous samples that were expressed from the RNAseq data. Therefore, we considered it unlikely that higher coverage genomic data would have strongly benefitted

the ASE tests. Especially as hybrid individuals were sequenced to relatively high coverage for RNAseq analyses (30X).

Minor comments

Lines 150-151: Delmore references should be Delmore et al, right?

Change made (lines 152, 153).

Line 467: Provide information about the annotations of the assembly. How was the annotation created, what release is it and how many genes in total?

Details have been added (lines 484-488).

Line 522: Be more specific about the cleaning in picardtools. Remove duplicates and/or some other command?

Additional details added (line 560).

Line 834: It would have been good to add the detailed workflows for the reviewing process, as it would have made it clearer how the expression analyses actually had been performed.

We have created a github repo with basic workflows for our analyses (https://github.com/kdelmore/swth_rnaseq).

Table S4: It says in the table header that there are five brain regions, but there only seems to be data for three regions.

Change made.

REVIEWER COMMENTS

Reviewer #1 (Remarks to the Author):

This is a very clearly written, revised manuscript reporting the results of an investigation into the gene expression in five brain regions of migratory vs. non-migratory, and parental vs. hybrid, Swainson's thrushes. I believe the authors have addressed the previous round of reviews well. The work is an interesting contribution to behavioral genetics, and is likely to also be the foundation for some very interesting follow-up research.

Reviewer #2 (Remarks to the Author):

I’m happy to see that the authors have clarified the text and performed some of the suggested changes. However, I still have some concerns about the methods and the results:

1) Residual reference bias

My understanding now is that the genome was masked for fixed or nearly fixed variants (FST>=0.9). The reference genome could still have alleles that are much more common in one of the subspecies and that could potentially still have an impact on the mapping of reads from each subspecies. This could consequently generate exaggerated allele-specific expression values for some genes and explain the strong relationship between FST and ASE, even if there of course could also be a true biological explanation behind this pattern.

In the previous response, the authors brought up that only uniquely mapped reads in both genomes were selected and then used to calculate FST for the resequenced samples. This is not really what I had in mind when speaking about reference bias. Rather, I was more concerned about how the RNAseq reads are mapping from the different genomes. Extrapolating from reference bias in resequenced reads are difficult because another mapper is used for RNAseq and these reads will often be mapped across exons.

Hence, I still think it will be worthwhile to make a more stringent masking of the reference genome to make the results more robust. For example, by masking all SNPs found in the resequencing data or at least using a considerably lower FST threshold (say FST 0.1 or 0.2). Alternatively, try using a software such as WASP. These analyses would obviously mean some extra work, but the analyses are straightforward and I think that they will make the conclusions more robust.

2) Treatment of allele counts

My understanding from the text and the equations is now that reads for each allele were summarized for each individual and for each gene. To provide a combined value across the samples, the coastal and inland alleles were averaged across samples. If this is the case, I still don't think that it's a correct method to analyze the data.

The coastal and inland alleles for a specific sample are paired – they are dependent on the expression levels of that gene in that specific sample. If one want to average across the samples, one should first calculate the ratio for each sample separately and then take an average of this value. Other studies using this method seem to require a minimum number of reads per sample for it to have a reliable ratio.

Now it is still possible that the method that I think the authors have been using (averaging read counts rather than ratios) could, at least under some circumstances, pick up allele-specific expression. This seems to be the case when analyzing all the hybrids (assuming no residual reference bias). It would probably work well when there is similar and widespread expression among the samples. However, it would not be technically correct and there could be issues when there is more variability among the samples in terms of expression levels.

The authors refer to other studies that are claimed to have used the same approach. I agree to some part with this, but not entirely. After having read Hu et al 2022 (Cell Reports), my understanding is that they didn't sum reads across samples, but contrasted pairs of samples originating from two different conditions. They had different pairs and performed the same statistical test for each pair and then combined the significance from the pairwise tests. This makes sense, because for each contrast, they have the allele counts from the same sample paired.

So, in the end, I would argue a more ratio-focused approach, where the counts within a specific sample are first contrasted against each other before obtaining a value across samples.

Note, if this is the approach that has been used, it has to be communicated way clearer than has been done so far in the text and in the equations.

3) Comparing G and GxE results

I agree that there are differences when looking into the results when comparing ASE in all hybrids versus contrasting across hybrids and conditions. These differences could very well have a biological explanation in terms of cis- and trans-regulation.

That said, it is important to ensure that there are no methodological issues that could give rise to different results between the G and GxE analyses.

One such thing, which I brought up earlier, is if the two groups differ in terms of hybrids and backcrosses. It has now been statistically checked that there is no difference in these measurements. There is also no apparent overall effect of coverage on genetic ancestry and heterozygosity. To make the data more transparent, it would be good to add both the ancestry value and heterozygosity for each hybrid to Table S1. Another thing to clarify the composition of each group would be to color the dots Figure S1 according to condition. In this way, one could better see how different these groups are in the joint distribution of these parameters.

I was also a little bit surprised about the lack of relationship between coverage and heterozygosity. Is it possible that most of this effect is driven by the ancestry of the sample (hybrid vs more parental) rather than the actual coverage? For example, do samples with an ancestry around 0.5 that have very low heterozygosity also have a very low coverage and those that have much higher heterozygosity have a higher coverage? If this is the case, there may be more F1s in this set of samples than is evident from Figure S1, which would be a good thing for the ASE analyses.

Another thing is how reliable the differences in ASE is between the two conditions. That is, for the genes with most extreme ratios in ASE between the conditions, do samples in both of the conditions have sufficiently high coverage that ASE within each group could be reliably estimated. For example, the approach used by Hu et al takes this into account by using the 2x2 table of read counts with a Fisher's exact test. This test cannot easily be used for this study, but is there some other way to make sure that the differences between the groups in ASE are reliable?

Unless there is something better, one could for example have calculated the ratios for each sample for each condition (and for each gene), used some form minimal requirements in terms of read coverage for each sample (to get reliable sample estimates) and a minimum number of samples fulfilling this requirement (for example 3) to get a reliable estimate for each condition. Then it would be possible to contrast the ratios between the conditions using a t-test or similar test for two groups.

4) ASEP analysis

The authors use an alternative method (ASEP) to quantify ASE in each of the two conditions and find that there is a strong overlap in the set of significant genes between the conditions. They argue that if cis-regulation would have been important, we would have expected to see little sharing between the

significant gene sets between the conditions. With the observed extensive sharing of genes, they thus conclude that these results further support their idea of trans-regulation being the most important factor in driving expression differences across conditions in hybrids.

However, another and, in my opinion, more plausible explanation is simply that the overlap between the conditions is picking up the genes that show universal (or at least widespread) allele-specific expression in the hybrids. These are the genes that were picked up in the first analysis, when using all of the hybrids. In any case, this observation alone is not sufficient to say that it is supporting a trans-regulation but it is also not saying that it cannot be the case for some of the genes. It is just not informative enough to provide support to this hypothesis.

5) Github page

While the shell scripts and software commands deposited on the github page look good (at least nothing obviously wrong to me), there are several comments that things should be added to the text. Indeed, there seems be quite a bit of missing information regarding for example the ASE analyses. I understand that this page have been made in a hurry, but it is important to have this information available for reviewers, in particular when there are previous comments about the methods. Also, before a future publication, I would be great if there could be a little more text in the general readme file, just giving a brief overview of the main files and folders.

Minor comments:

Line 479-480. Regarding the coverage, it is a little bit unclear what it means for the RNAseq samples. Are these the values they would correspond to if there were evenly spread out over the genome? That is, what would they would mean for resequenced data. For RNAseq, this is not really true, because they originate from a smaller part of the genome. For RNAseq I often see number of reads, instead of coverage. Is it possible to clarify? Ideally, it would also be good to give the number of mapped reads for each of the samples in Table S1. This could easily be obtained by using for example samtools on the bam files.

Reviewer #1 (Remarks to the Author):

This is a very clearly written, revised manuscript reporting the results of an investigation into the gene expression in five brain regions of migratory vs. non-migratory, and parental vs. hybrid, Swainson's thrushes. I believe the authors have addressed the previous round of reviews well. The work is an interesting contribution to behavioral genetics, and is likely to also be the foundation for some very interesting follow-up research.

Thank you for your kind remarks and support.

Reviewer #2 (Remarks to the Author):

I'm happy to see that the authors have clarified the text and performed some of the suggested changes. However, I still have some concerns about the methods and the results:

1) Residual reference bias

My understanding now is that the genome was masked for fixed or nearly fixed variants (FST>=0.9). The reference genome could still have alleles that are much more common in one of the subspecies and that could potentially still have an impact on the mapping of reads from each subspecies. This could consequently generate exaggerated allele-specific expression values for some genes and explain the strong relationship between FST and ASE, even if there of course could also be a true biological explanation behind this pattern.

In the previous response, the authors brought up that only uniquely mapped reads in both genomes were selected and then used to calculate FST for the resequenced samples. This is not really what I had in mind when speaking about reference bias. Rather, I was more concerned about how the RNAseq reads are mapping from the different genomes. Extrapolating from reference bias in resequenced reads are difficult because another mapper is used for RNAseq and these reads will often be mapped across exons. Hence, I still think it will be worthwhile to make a more stringent masking of the reference genome to make the results more robust. For example, by masking all SNPs found in the resequencing data or at least using a considerably lower FST threshold (say FST 0.1 or 0.2). Alternatively, try using a software such as WASP. These analyses would obviously mean some extra work, but the analyses are straightforward and I think that they will make the conclusions more robust.

Thank you for expanding on your original comment. After running the requested analysis, we have decided to continue using our original threshold for several reasons.

First, as requested by the reviewer, we have rerun mapping with our RNAseq data masking SNPs with FST > 0.1. Data on the number of reads that map under both thresholds (our original threshold where reads were mapped at FST > 0.9 and the new threshold of 0.1) can be found in Table S1. There is no significant difference in the number of reads mapped under these thresholds. More reads do map with FST > 0.9 (as you'd expect since fewer bases are masked under this threshold) but it is a VERY small (between 0.02% and 0.6% of reads [4828-24676 reads]) and insignificant difference (linear model with # uniquely mapped reads as response and threshold as predictor, $F_{1,64} = 4x10^{-5}$, p-value = 0.99). We have referenced this analysis in the text (lines 494-498). Note, there are no differences between the populations (coastal, inland and hybrid) and seasons in the number of reads that map to the reference genome (linear model with population*season as the predictor variables, all p-values > 0.55).

Second, we do not expect much mapping bias in our system – we are dealing with two subspecies that exhibit very little genomic differentiation (genome-wide FST = 0.026).

Finally, if we understand correctly, the reviewer is concerned that our findings of (1) ASE in subspecies only genes (i.e., that cis-regulatory changes generated DE at this level) and (2) elevated FST at SNPs near these genes are not independent. Instead, they are concerned mapping bias is generating ASE (allowing more inland alleles to align) and this happens where FST is higher, creating what looks like ASE at areas with elevated FST. That does not appear to be the case as there is no bias towards inland alleles in our analyses; for example, focus on data from the CNH, we have ~180K total counts for SNPs diagnostic of the inland subspecies and 172K for the coastal subspecies. Furthermore, the distribution of gene log fold change (LFC) values for the hybrids demonstrates that there is no bias towards inland subspecies expression. Below is a histogram and density plot of the LFC values for each gene investigated in the subspecies comparison for CNH (i.e., values from Figure 4a). We have referenced these analyses in the text (lines 494-498).

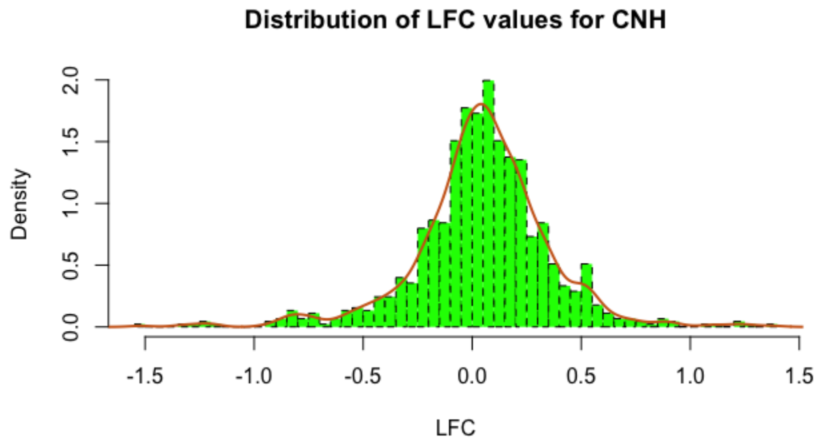

In sum, there are several reasons why we do not think it is necessary use a different threshold for masking (no significant/substantial difference in mapping, low genomic divergence, and no bias towards inland alleles at loci showing ASE). We thank the reviewer for this comment as we believe the additions they requested have strengthened the paper.

2) Treatment of allele counts

My understanding from the text and the equations is now that reads for each allele were summarized for each individual and for each gene. To provide a combined value across the samples, the coastal and inland alleles were averaged across samples. If this is the case, I still don't think that it's a correct method to analyze the data.

The coastal and inland alleles for a specific sample are paired – they are dependent on the expression levels of that gene in that specific sample. If one wants to average across the samples, one should first calculate the ratio for each sample separately and then take an average of this value. Other studies using this method seem to require a minimum number of reads per sample for it to have a reliable ratio.

Now it is still possible that the method that I think the authors have been using (averaging read counts rather than ratios) could, at least under some circumstances, pick up allele-specific expression. This

seems to be the case when analyzing all the hybrids (assuming no residual reference bias). It would probably work well when there is similar and widespread expression among the samples. However, it would not be technically correct and there could be issues when there is more variability among the samples in terms of expression levels.

The authors refer to other studies that are claimed to have used the same approach. I agree to some part with this, but not entirely. After having read Hu et al 2022 (Cell Reports), my understanding is that they didn't sum reads across samples, but contrasted pairs of samples originating from two different conditions. They had different pairs and performed the same statistical test for each pair and then combined the significance from the pairwise tests. This makes sense, because for each contrast, they have the allele counts from the same sample paired.

So, in the end, I would argue a more ratio-focused approach, where the counts within a specific sample are first contrasted against each other before obtaining a value across samples.

Note, if this is the approach that has been used, it has to be communicated way clearer than has been done so far in the text and in the equations.

Thank you for expanding on your original comment. After running the requested analysis, we have chosen to keep our analysis of allele counts as is for several reasons.

First, we conducted the analysis requested, calculating a ratio for each individual before taking an average across all individuals. Our results remain very similar. For example, focusing on data for CNH, the correlation coefficient between values obtained in our original approach and that suggested by the reviewer was 0.96 (p-value < 0.0001; see Figure below). In addition, the relationship between log fold changes (LFC) estimated for pure and hybrid forms does not change with the new values (e.g., focusing on genes differentially expressed between the subspecies regardless of season [Figure 4a] the correlation coefficient goes from 0.54 [95% confidence interval = 0.49-0.58] to 0.56 [0.51 – 0.60]). We have referenced this analysis in the text (lines 530-533).

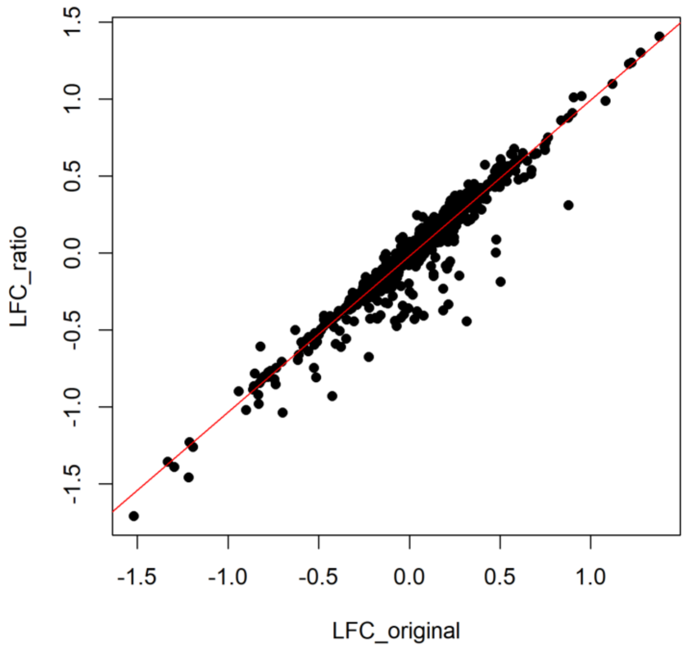

Second, the main objective of this analysis was simply to understand the evolutionary mechanisms that underlie patterns of differential expression in our system. This was the same question Hu et al. (2022) asked in Figure 2G of their paper and those authors used the same approach we employed in our study, summarizing counts across individuals heterozygous of subspecies. Hu et al. (2022) expanded on their original finding, using a paired design to identify specific genes showing ASE, but that is not a question we asked in our study. As noted in our original response, Hu et al. (2022) is not the only paper to use the approach we described here – York et al. (2018) also used the approach and a recent paper by Mack et al. (2023 Mol Biol Evol 40) did as well.

Finally, we do not think it is correct to state that coastal and inland alleles are paired in a specific sample; the number of inland alleles does not depend on the number of coastal alleles at a locus (and vice versa). Total allele count at a given gene is simply the sum of alleles (inland or coastal) at that gene. If coverage were low at a particular gene, we might lack confidence in our allele counts but we required each gene to have 10 reads covering it in order to be included in our analysis.

Hopefully the above arguments make it clear that our approach was appropriate for this analysis. Nevertheless, we agree with the reviewer that rationale for our approach needed more clarification. Accordingly, we have edited the text (lines 172-175), to be clear that we looked at the correlation between ASE and subspecies differences in expression to gain insight into the evolutionary mechanisms, rather than identifying individual candidate genes with significant ASE. We have also highlighted the fact that each gene had to have 10 reads covering it in order to be included in our analysis (lines 528-530) and noted that we obtain similar results when we estimate ratios for each individual first (lines 530-533). As noted above, we thank the reviewer for their careful review of this analysis.

3) Comparing G and GxE results

I agree that there are differences when looking into the results when comparing ASE in all hybrids versus

contrasting across hybrids and conditions. These differences could very well have a biological explanation in terms of cis- and trans-regulation. That said, it is important to ensure that there are no methodological issues that could give rise to different results between the G and GxE analyses.

One such thing, which I brought up earlier, is if the two groups differ in terms of hybrids and backcrosses. It has now been statistically checked that there is no difference in these measurements. There is also no apparent overall effect of coverage on genetic ancestry and heterozygosity. To make the data more transparent, it would be good to add both the ancestry value and heterozygosity for each hybrid to Table S1. Another thing to clarify the composition of each group would be to color the dots Figure S1 according to condition. In this way, one could better see how different these groups are in the joint distribution of these parameters.

As requested, ancestry and heterozygosity have been added to Table S1 and dots in Figure S1 have been colored according to migratory state.

I was also a little bit surprised about the lack of relationship between coverage and heterozygosity. Is it possible that most of this effect is driven by the ancestry of the sample (hybrid vs more parental) rather than the actual coverage? For example, do samples with an ancestry around 0.5 that have very low heterozygosity also have a very low coverage and those that have much higher heterozygosity have a higher coverage? If this is the case, there may be more F1s in this set of samples than is evident from Figure S1, which would be a good thing for the ASE analyses.

When we limit the data to birds with ancestry values between 0.25 and 0.75, we find no relationship between heterozygosity and coverage (p-value = 0.32). As noted in our response to the first comment, these are subspecies with limited genomic divergence. As outlined in our original response, we have also limited reference bias with the WGS data by aligning data to both references and limiting our analyses to reads that map to both references. Accordingly, we would not expect to find this relationship.

Another thing is how reliable the differences in ASE is between the two conditions. That is, for the genes with most extreme ratios in ASE between the conditions, do samples in both of the conditions have sufficiently high coverage that ASE within each group could be reliably estimated. For example, the approach used by Hu et al takes this into account by using the 2x2 table of read counts with a Fisher's exact test. This test cannot easily be used for this study, but is there some other way to make sure that the differences between the groups in ASE are reliable?

As noted above, Hu et al. (2022) used this analysis to identify specific genes whose expression levels are controlled by cis-regulatory changes. We are not asking that question in our manuscript. In addition, Hu et al. (2022) use the paired design because they have data from siblings and thus, have to controlled for shared genetic backgrounds. And finally, we do require each gene to have 10 allele coverage to be included in our analyses. We have added the details to the manuscript (lines 172-175; lines 528-530).

Unless there is something better, one could for example have calculated the ratios for each sample for each condition (and for each gene), used some form minimal requirements in terms of read coverage for each sample (to get reliable sample estimates) and a minimum number of samples fulfilling this requirement (for example 3) to get a reliable estimate for each condition. Then it would be possible to

contrast the ratios between the conditions using a t-test or similar test for two groups.

This appears to be the same concern/suggestion brought up in comment #2. Please refer to our response above.

4) ASEP analysis

The authors use an alternative method (ASEP) to quantify ASE in each of the two conditions and find that there is a strong overlap in the set of significant genes between the conditions. They argue that if cis-regulation would have been important, we would have expected to see little sharing between the significant gene sets between the conditions. With the observed extensive sharing of genes, they thus conclude that these results further support their idea of trans-regulation being the most important factor in driving expression differences across conditions in hybrids.

However, another and, in my opinion, more plausible explanation is simply that the overlap between the conditions is picking up the genes that show universal (or at least widespread) allele-specific expression in the hybrids. These are the genes that were picked up in the first analysis, when using all of the hybrids. In any case, this observation alone is not sufficient to say that it is supporting a trans-regulation but it is also not saying that it cannot be the case for some of the genes. It is just not informative enough to provide support to this hypothesis.

This analysis was added in response to the reviewers' previous comments. In response to the present comment, we have removed this analysis from the manuscript.

5) Github page

While the shell scripts and software commands deposited on the github page look good (at least nothing obviously wrong to me), there are several comments that things should be added to the text. Indeed, there seems be quite a bit of missing information regarding for example the ASE analyses. I understand that this page have been made in a hurry, but it is important to have this information available for reviewers, in particular when there are previous comments about the methods. Also, before a future publication, I would be great if there could be a little more text in the general readme file, just giving a brief overview of the main files and folders.

We agree that more information about analyses was needed. We have updated the github repo in response (https://github.com/kdelmore/swth_rnaseq).

Minor comments:

Line 479-480. Regarding the coverage, it is a little bit unclear what it means for the RNAseq samples. Are these the values they would correspond to if there were evenly spread out over the genome? That is, what would they would mean for resequenced data. For RNAseq, this is not really true, because they originate from a smaller part of the genome. For RNAseq I often see number of reads, instead of coverage. Is it possible to clarify? Ideally, it would also be good to give the number of mapped reads for each of the samples in Table S1. This could easily be obtained by using for example samtools on the bam files.

We have added this information to Table S1 as requested.

REVIEWERS' COMMENTS

Reviewer #2 (Remarks to the Author):

The authors have performed the revisions and analyses I recommended for the previous version of the manuscript.

I’m happy to see that the main results are still supported and that the study design and analyses are now more transparent.

Reviewer #2 (Remarks to the Author):

The authors have performed the revisions and analyses I recommended for the previous version of the manuscript.

I'm happy to see that the main results are still supported and that the study design and analyses are now more transparent.

Thank you.
